# Neural Correlates of Facial Mimicry: Simultaneous Measurements of EMG and BOLD Responses during Perception of Dynamic Compared to Static Facial Expressions

**DOI:** 10.3389/fpsyg.2018.00052

**Published:** 2018-02-06

**Authors:** Krystyna Rymarczyk, Łukasz Żurawski, Kamila Jankowiak-Siuda, Iwona Szatkowska

**Affiliations:** ^1^Department of Experimental Psychology, Institute of Cognitive and Behavioural Neuroscience, SWPS University of Social Sciences and Humanities, Warsaw, Poland; ^2^Laboratory of Psychophysiology, Department of Neurophysiology, Nencki Institute of Experimental Biology of Polish Academy of Sciences, Warsaw, Poland

**Keywords:** facial mimicry, EMG, fMRI, mirror neuron system, emotional expressions, dynamic, happiness, anger

## Abstract

Facial mimicry (FM) is an automatic response to imitate the facial expressions of others. However, neural correlates of the phenomenon are as yet not well established. We investigated this issue using simultaneously recorded EMG and BOLD signals during perception of dynamic and static emotional facial expressions of happiness and anger. During display presentations, BOLD signals and zygomaticus major (ZM), corrugator supercilii (CS) and orbicularis oculi (OO) EMG responses were recorded simultaneously from 46 healthy individuals. Subjects reacted spontaneously to happy facial expressions with increased EMG activity in ZM and OO muscles and decreased CS activity, which was interpreted as FM. Facial muscle responses correlated with BOLD activity in regions associated with motor simulation of facial expressions [i.e., inferior frontal gyrus, a classical Mirror Neuron System (MNS)]. Further, we also found correlations for regions associated with emotional processing (i.e., insula, part of the extended MNS). It is concluded that FM involves both motor and emotional brain structures, especially during perception of natural emotional expressions.

## Introduction

Facial mimicry (FM) is an unconscious and unintentional automatic response to the facial expressions of others. Numerous studies have shown that observing the emotional states of others leads to congruent facial muscle activity. For example, observing angry facial expressions can result in enhanced activity in the viewer's muscle responsible for frowning (CS), while viewing happy images leads to Increased activity in the facial muscle involved in smiling (ZM), and decreased activity of the CS (Hess et al., 1998; Dimberg and Petterson, 2000). However, it has recently been suggested that FM may not be an exclusive automatic reaction but rather a multifactorial response dependent on properties such as stimulus modality (e.g., static or dynamic) or interpersonal characteristics (e.g., emotional contagion susceptibility) (for review see Seibt et al., 2015).

There are two main psychological approaches trying to explain the mechanisms of FM. One of these is the perception-behavior link model which assumes perception and execution of a specific action show a certain overlap (Chartrand and Bargh, 1999). According to the theory, mere perception of the emotional facial expressions of others automatically evokes the same behavior in the perceiver, and the facial expression is copied spontaneously (Chartrand and Bargh, 1999; Dimberg et al., 2000). This notion was supported by recent evidence from neuroimaging literature showing that both the perception and execution of facial emotional expressions engage overlapping brain structures, such as the inferior frontal gyrus (IFG) and inferior parietal lobule (IPL) (Carr et al., 2003; Rizzolatti and Craighero, 2004; Iacoboni and Dapretto, 2006), being regions constituting the classical mirror neuron system (MNS). An example of empirical support for this assumption can be found in a study involving patients with Parkinson's disease where patients demonstrated difficulties with both execution of emotional expression and identification of emotions (Livingstone et al., 2016). Another approach describes FM as a consequence of contagion to the emotional states of others (Hatfield et al., 1993; Bastiaansen et al., 2009). In other words, the observation of other's emotional facial expressions triggers corresponding emotions in the observer. It is suggested that contagion occur due to direct activation of neural substrate, which is involved in the experience of the observed emotion (Wicker et al., 2003). Those emotional-related brain structures, i.e. insula and amygdala, among others related to extended MNS, were activated during both the observation and execution of emotional facial expressions (Carr et al., 2003; van der Gaag et al., 2007; Kircher et al., 2013).

It is worth noting that most of what we know about the neural correlates of automatic FM has been derived from functional neuroimaging studies during the passive viewing or the imitation of emotional facial displays presented to subjects. Direct investigation of the neural correlates of FM, such as simultaneous measurement of BOLD responses (using functional magnetic resonance imaging, fMRI) and facial muscular reactions (using electromyography, EMG) may contribute to improved understanding of the neural basis of FM. To date, only one study (Likowski et al., 2012) has examined the brain structures involved in the occurrence of automatic facial reactions by simultaneously measuring BOLD and facial EMG signals in an MRI scanner. These investigators found that automatic and spontaneous FM of happiness, sadness, and anger displays led to activation of a prominent part of the classic MNS (i.e., the IFG), as well areas responsible for emotional processing (i.e., the insula). They concluded that the perception of emotional facial expressions activated a variety of structures presumed to belong to the classic and extended MNS, but only a small number were correlated with the magnitude of FM. It is currently unknown whether the perception of real, dynamic emotional facial expressions rather than static avatars, used in the study (Likowski et al., 2012), would reveal more associations between the strength of the FM reactions and regional brain activation. Importantly, recent neuroimaging studies (Trautmann et al., 2009; Arsalidou et al., 2011; Kessler et al., 2011) have found that the perception of dynamic emotional stimuli, in comparison to static stimuli, engages a widespread activation pattern that involves parts of the MNS, including the IFG (Sato et al., 2004, 2015; Kessler et al., 2011) and other emotion-related structures like the amygdala and insula (Kilts et al., 2003; Trautmann et al., 2009). Indeed, it has been demonstrated that dynamic emotional facial expressions can improve emotion recognition of subtle facial expressions (Ambadar et al., 2005; Trautmann et al., 2009), enhance emotional arousal (Sato and Yoshikawa, 2007), and elicit stronger FM than static presentations (Weyers et al., 2006; Sato et al., 2008; Rymarczyk et al., 2011). In light of these studies, determining which brain structures are involved in automatic, spontaneous FM could be addressed, at least in part, by simultaneous measurement of facial muscular activity (EMG) and the BOLD responses (fMRI) during passive perception of real, dynamic emotional facial expressions.

In the present study, we simultaneously recorded EMG and BOLD signals during the perception of realistic dynamic and static emotional facial expressions. We measured facial EMG responses from three muscles, the ZM, CS, and OO, while participants passively viewed happy, angry, and neutral displays. Following earlier research, we measured facial muscle activity over the cheek region ZM involved in smiling and over the brow CS region responsible for frowning (e.g., Andréasson and Dimberg, 2008). The activity over the eye OO, typically linked with true joy, smile expression (Hess and Blairy, 2001; Hess and Bourgeois, 2010; Korb et al., 2014) was also measured. It was proposed that contraction of OO transforms a non-Duchenne into a Duchenne smile (Ekman and Rosenberg, 2012). Other researchers suggested that contractions of OO could be additionally indicative for the negative signal value of anger configurations, discomfort-pain or distress-cry situations (Russell and Fernandez-Dols, 1997). Based on previous studies (van der Gaag et al., 2007; Jabbi and Keysers, 2008; Likowski et al., 2012), we anticipated that motor and emotional brain structures would be responsible for differences in automatic FM during perception of dynamic compared to static displays. We examined which of the classic and extended MNS regions showed a relationship with the strength of facial reactions. Furthermore, since dynamic facial expressions constitute a more powerful medium for emotional communication than static presentations, we anticipated that regional brain activation and muscle responses would be more pronounced for dynamic emotional facial expressions. We predicted that presentations of dynamic happy facial expressions would engage brain areas associated with the representation of pleasant feelings and reward (such as the basal ganglia structures, in particular the nucleus accumbens) and would correlate with increased activity of the ZM and OO muscles. For dynamic facial expression anger, we predicted co-activation of limbic structures (i.e., amygdala), proposed to be involved in the automatic detection of evolutionary threats (van der Zwaag et al., 2012), would be associated with CS activity.

## Methods

### Subjects

Forty-six healthy individuals (21 females, 26 males; mean age = 23.7 ± 2.5 years) participated in this study. The subjects had normal or corrected to normal eyesight and none of them reported neurological diseases. This study was carried out in accordance with the recommendations of Ethics Committee at the University of Social Sciences and Humanities with written informed consent from all subjects. All subjects gave written informed consent in accordance with the Declaration of Helsinki. The protocol was approved by the Ethics Committee at the University of Social Sciences and Humanities. An informed consent form was signed by each participant after the experimental procedures had been clearly explained. After the scanning session, subjects were informed about the aim of the study.

### Facial stimuli and apparatus

We used videos and static emotional pictures illustrating forward-facing facial expressions of happiness and anger taken from The Amsterdam Dynamic Facial Expression Set (van der Schalk et al., 2011). Additionally, we included neutral conditions (no visible emotional facial expression) presented as static and dynamic displays. Dynamic stimuli clips of three males and females were used (F01, F03, F09, M03, M07, M11). Each character presented happy, angry, and neutral facial expressions and participants observed only one type of expression at a time (as a photo or a video). In the case of neutral dynamic condition, the motion could be observed because characters were either closing their eyes or slightly changing the position of their head. Each stimulus in the neutral static condition presented one frame from the dynamic video clip, used in the neutral dynamic condition. Stimuli were 576 pixels in height and 720 pixels in width. Expressions were presented on a gray background. All procedures were controlled using Presentation® software running on a computer with Microsoft Windows operating system and were displayed on a 32-inch NNL LCD MRI-compatible monitor (1,920 × 1,080 pixels resolution; 32 bit color rate; 60 Hz refresh rate) from a viewing distance of approximately 140 cm.

### EMG acquisition

Data were recorded using an MRI-compatible BrainCap (Brain Products) consisting of 3 bipolar and one reference electrode with a diameter of 2 mm and filled with electrode paste. The electrodes were positioned in pairs over three muscles—the CS, ZM and OO on the left side of the face (Cacioppo et al., 1986; Fridlund and Cacioppo, 1986). A reference electrode, 2 mm in diameter, was attached to the forehead. Before the electrodes were attached, the skin was cleaned with alcohol and a thin coating of electrode paste was applied. This procedure was repeated until electrode impedance was reduced to 5 kΩ or less. The digitized EMG signals were recorded using a BrainAmp MR plus ExG amplifier and BrainVision Recorder. The hardware low-pass filtered the signal at 250 Hz. Finally, data was digitized with a sampling rate of 5 kHz, and stored on a computer running MS Windows 7 for offline analysis.

### Image acquisition

MRI acquisition was acquired on a Siemens Trio 3 T MR-scanner equipped with 12-channel phased array head coil. Functional MRI images were registered using T2^*^-weighted EPI gradient-echo pulse sequence with the following parameters: TR = 2,000 ms, TE = 25 ms; 90° flip angle, FOV = 250 mm, matrix = 64 × 64, voxel size = 3.5 × 3.5 × 3.5 mm, interleaved even acquisition, slice thickness = 3.5 mm, 39 slices.

### Procedure

Each volunteer was introduced to the experimental procedure and signed a consent form. To conceal the true purpose, facial electromyography recordings, participants were told that sweat gland activity was being recorded while watching the faces of actors selected for commercials by an external marketing company. Following the attachment of the electrodes of the FaceEMGCap-MR, participants were reminded to carefully observe the actors presented on the screen and were positioned in the scanner. The subjects were verbally encouraged to feel comfortable and behave naturally.

**Figure 1:**
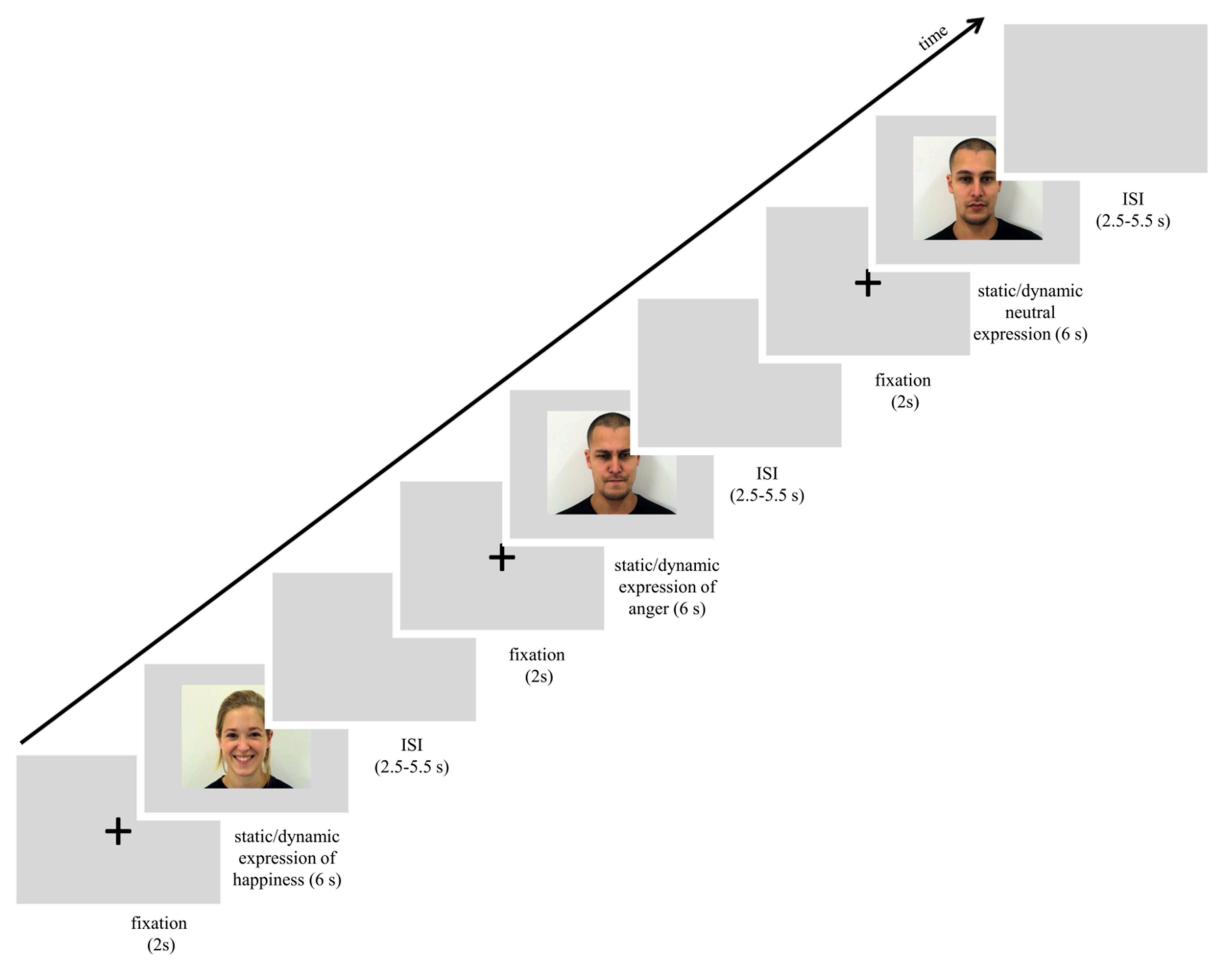
Scheme of procedure used in the study.

The scanning session started with a reminder of the subject's task. In the session subjects were presented with 72 trials that lasted approximately 15 min. Each trial started with a white fixation cross, 80 pixels in diameter, which was visible for 2 s in the center of the screen. Next, one of the stimuli with a facial expression (happy, angry or neutral, each presented as static image or dynamic video clip) was presented for 6 s. The expression was followed by a blank gray screen presented for 2.75–5.25 s (see Figure 1). All stimuli were presented in the center of the screen. In summary, each stimulus was repeated once, for a total of 6 presentations within a type of expression (e.g., 6 dynamic presentations of happiness). The stimulus appeared in an event-related manner, pseudo-randomized trial by trail with constraints in randomization: no facial expression from the same actor, and no more than 2 actors of the same sex or the same emotion were presented consecutively. In total, 6 randomized event-related sessions with introduced constraints were balanced between subjects.

### Data analysis

#### EMG analysis

Pre-processing was carried out using The BrainVision Analyser 2 (version 2.1.0.327). First, EPI gradient-echo pulse artifacts were removed using the average artifact subtraction AAS method (Allen et al., 2000) implemented in the Analyser based on the sliding average calculation and consisting of 11 consecutive functional volumes marked in the data logs. A successful AAS method is possible due to synchronization hardware and markers that were created by the triggers received from the MR system. Next, data were filtered at 30 Hz high-pass and 500 Hz low-pass filters. After rectification and integration over 125 ms, the signal was resampled to 10 Hz. Artifacts related to EMG were detected in two ways. Firstly, when single muscle activity was above 8 μV at baseline (visibility of the fixation cross) (Weyers et al., 2006; Likowski et al., 2008, 2011), the trial was classified as an artifact and excluded from further analysis (*M* = 3,8 trials per participant were excluded). All remaining trials were blind-coded and visually checked for artifacts. Later, trials were baseline corrected such that the EMG response was measured as the difference of averaged signal activity between the stimuli duration (6 s) and baseline period (2 s). Finally, the signal was averaged for each condition, for each participant and was imported to SPSS 21 for statistical analysis.

For testing differences in EMG responses, a two-way repeated-measures ANOVA with two within-subjects factors (expression: happiness, anger, neutral; and stimulus modality: dynamic, static) were used. Separate ANOVAs were calculated for responses from a single muscle and reported with a Bonferroni correction. In order to confirm that EMG activity changed from baseline and FM occurred, the EMG data of each significant effect were tested for a difference from zero (baseline) using one-sample, two-tailed *t*-tests.

#### Image processing and analysis

Image processing and analysis were carried out using SPM12 (6470) run in MATLAB 2013b (The Mathworks Inc., 2013). Functional images were motion-corrected and co-registered to the mean functional image. Brain structural images were segmented into different tissue classes—gray matter, white matter, and non-brain (cerebrospinal fluid, skull) using the segmentation module. Next, a DARTEL algorithm was used to create a study-specific template for all participants based on segmented structural images. The template was later affine registered to the MNI space, and the functional images were warped to this template and resliced to 2 × 2 × 2 mm isotropic voxels to be later smoothed with an 8 × 8 × 8 mm full-width at half maximum Gaussian kernel. Single subject design matrices included six experimental conditions (dynamic: happiness, anger, neutral; and static: happiness, anger, neutral) that were modeled with standard hemodynamic response function and other covariates produced by Artifact Detection Toolbox (ART) that included head movements and other parameters excluding the artificial fMRI signal. Later, the same sets of contrasts of interest were calculated for each subject and used in group level analysis (i.e., one-sample *t*-test) for statistical Regions of Interest (ROIs) analysis. The analysis was performed using the MarsBar toolbox (Brett et al., 2002) specifically for the separate ROIs. Anatomical region of interest masks were created with the WFU Pickatlas (Wake Forest University, 2014) (primary motor, premotor cortex, IPL, BA44, BA45, amygdala, ACC, insula, caudate head, putamen, nucleus accumbens, globus pallidus), and SPM Anatomy Toolbox (Eickhoff, 2016) [MT+/V5, primary somatosensory cortex (Areas 1, 2, 3a, 3b)]. The STS and pre-SMA ROIs were based on activation peaks from the literature (Van Overwalle, 2009) and a meta-analysis (Kohn et al., 2014), and were defined as an overlapping set of peaks with a radius of 8 mm each. The data were extracted as mean values of each ROI and statistics of brain activity were reported with Bonferroni correction applied to the data (i.e., *p*-value divided by number of ROIs).

#### Correlation analysis

Pearson correlation coefficients were calculated between selected contrasts of brain activity (happiness dynamic, happiness static, anger dynamic, anger static) and corresponding mimicry muscles activity in order to understand mutual relationship between brain activity and the facial muscle activity. Additionally, bias-corrected and accelerated (BCa) bootstrap 95% confidence intervals (samples = 1,000) were computed for Pearson correlation coefficients.

Each brain-ROI was represented by a single mean value (of all the voxels in anatomical atlas in each hemisphere). Each value was specific to each participant and ROI. Muscle activity was defined as average baseline corrected EMG trials of the same muscle and type. So the correlations were performed in pairs of variables of muscles (in specific conditions) and EMG responses, e.g., happiness_static_ZM with happiness_static_insulaRight.

## Results

### EMG measures

#### M. corrugator supercilii

**Figure 2:**
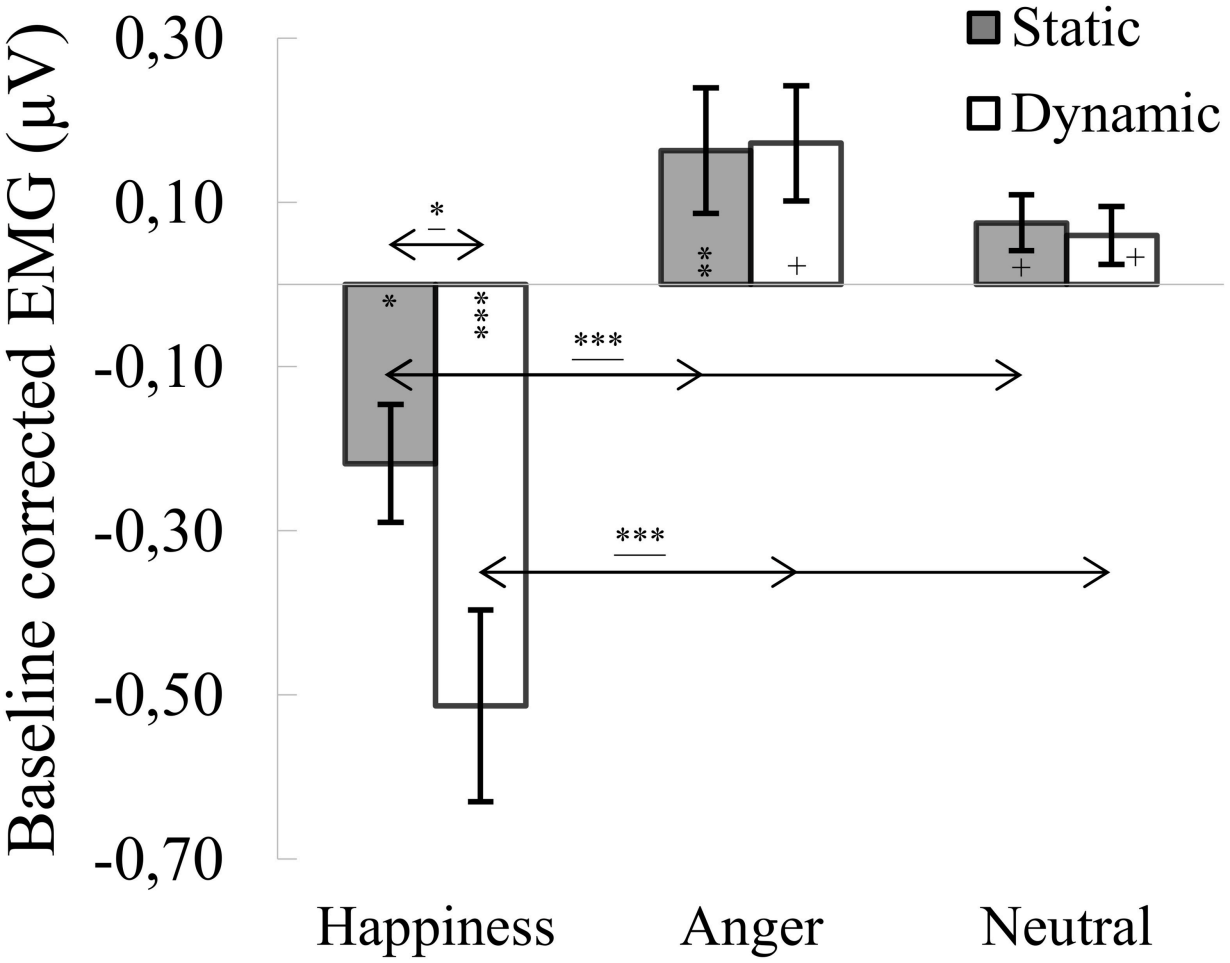
Mean (±SE) EMG activity changes and corresponding statistics for corrugator supercilii during presentation conditions. Asterisks with lines beneath indicate significant differences between conditions (simple effects) in EMG responses: ^*^p < 0.05, ^***^p < 0.001. Separate asterisks indicate significant differences from baseline EMG responses: ^+^*p* < 0.1, ^*^p < 0.05, ^**^p < 0.01, ^***^p < 0.001.

ANOVA showed a significant main effect of expression [*F*_(1.722_, _65.422)_ = 30.676, *p* < 0.001, η2 = 0.447], indicating that activity of the CS for happiness (*M* = −0.366, *SE* = 0.072) was lower than for angry [*M* = 0.168, *SE* = 0.067; *t*_(36)_ = 6.271, *p* < 0.001] and neutral expressions [*M* = 0.067, *SE* = 0.030; *t*_(36)_ = 6.186, *p* < 0.001]. The main effect of modality [*F*_(1, 38)_ = 4.020, *p* = 0.052, η2 = 0.096] approached significance with the activity of CS generally higher for static (*M* = 0.007, *SE* = 0.047) than dynamic [*M* = −0.094, *SE* = 0.050] facial expressions. The significant interaction of expression × modality [*F*_(1.389, 52.774)_ = 3.964, *p* = 0.039, η2 = 0.094] revealed that activity of the CS for dynamic and static happiness was lower than that for angry [*t*_(33)dynamic_ = 5.044, *p* < 0.001; *t*_(33)static_ = 5.219, *p* < 0.001] and neutral [*t*_(33)dynamic_ = 4.815, *p* < 0.001; *t*_(33)static_ = 3.959, *p* < 0.01] facial expressions, respectively (see Figure 2). The decrease of CS activity was higher for dynamic than static happiness conditions [*t*_(33)_ = 2.269, *p* = 0.029].

One-sample *t*-tests revealed significantly lower CS activity for dynamic [*t*_(40)_ = −4.595, *p* < 0.001] and static [*t*_(40)_ = −2.618, *p* = 0.012] happiness conditions, compared to baseline. CS responses for static anger [*t*_(41)_ = 2.724, *p* = 0.009] were higher than baseline. All other conditions were marginally higher than baseline [*t*_(39)anger_dynamic_ = 2.016, *p* = 0.051; *t*_(39)neutral_dynamic_ = 1.858, *p* = 0.071; *t*_(39)neutral_static_ = 1.827, *p* = 0.075].

#### M. orbicularis oculi

**Figure 3:**
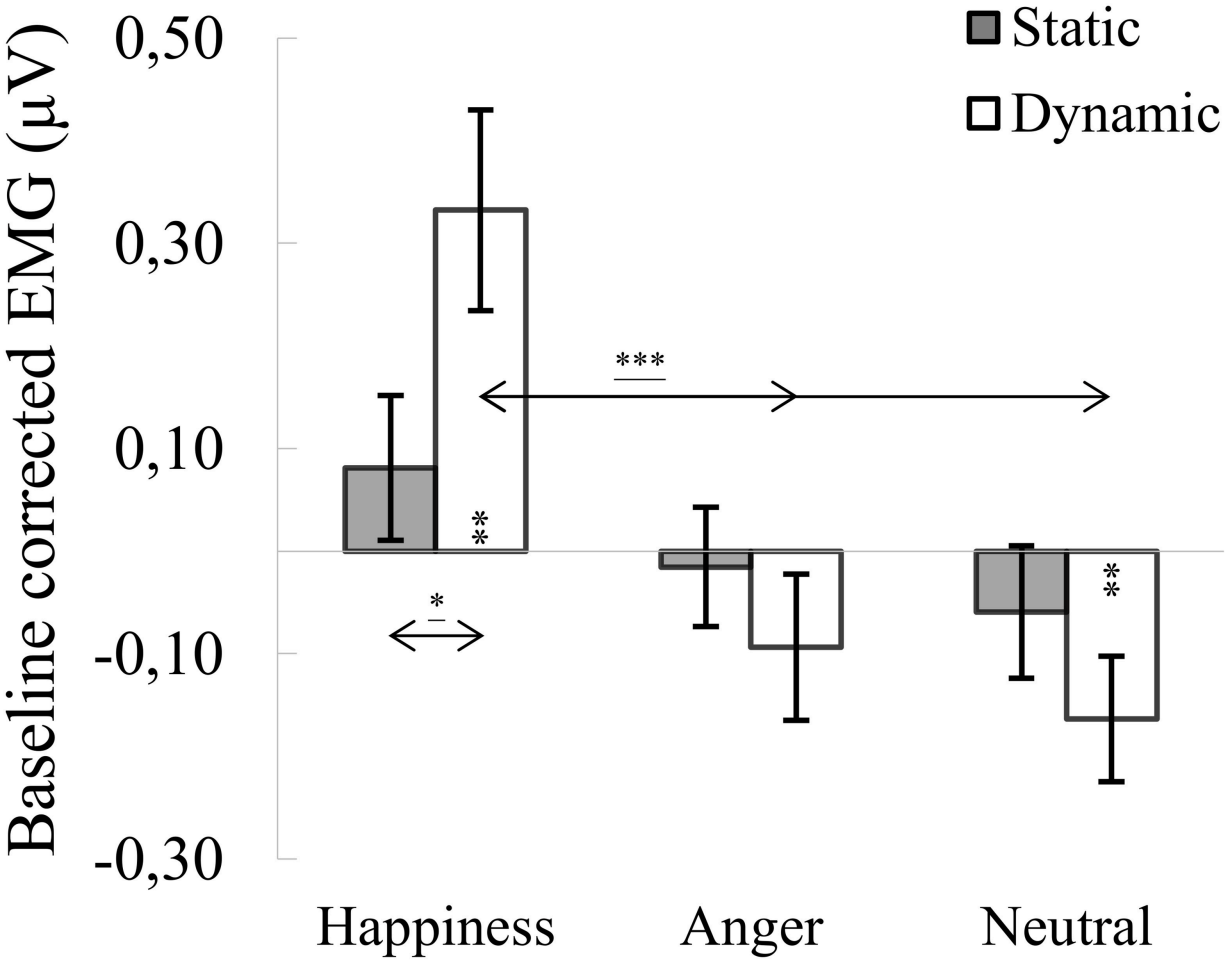
Mean (±SE) EMG activity changes and corresponding statistics for orbicularis oculi during presentation conditions. Asterisks with lines beneath indicate significant differences between conditions (simple effects) in EMG responses: ^*^p < 0.05, ^***^p < 0.001. Separate asterisks indicate significant differences from baseline EMG responses: ^**^p < 0.01.

ANOVA showed a significant main effect of expression [*F*_(2, 76)_ = 15.561, *p* < 0.001, η2 = 0.291], indicating that activity of the OO for happiness (*M* = 0.207, *SE* = 0.075) was higher than for angry [*M* = −0.054, *SE* = 0.055; *t*_(36)_ = 4.279, *p* < 0.001] and for neutral expressions [*M* = −0.111, *SE* = 0.045; *t*_(36)_ = 4.746, *p* < 0.001]. A significant expression × modality interaction [*F*_(1.688, 64.132)_ = 5.217, *p* = 0.011, η2 = 0.121] revealed that OO activity for dynamic expressions was higher than for static happiness [*t*_(33)_ = 3.099, *p* = 0.009]. Other observed differences included higher OO activity in the happiness dynamic compared to the angry dynamic [*t*_(33)_ = 4.303, *p* < 0.001] and neutral dynamic [*t*_(33)_ = 4.679, *p* < 0.001] facial expressions (see Figure 3).

One-sample *t*-tests revealed increased OO activity, compared to baseline, for dynamic happiness [*t*_(40)_ = 3.328, *p* = 0.002] and reduced activity for dynamic neutral [*t*_(40)_ = −2.862, *p* = 0.007] facial expressions. All other OO activities did not differ from baseline [*t*_(40)happiness_static_ = 1.032, *p* = 0.308; *t*_(39)anger_dynamic_ = −0.916, *p* = 0.365; *t*_(41)anger_static_ = −0.113, *p* = 0.911; *t*_(39)neutral_static_ = −0.857, *p* = 0.397].

#### M. zygomaticus major

**Figure 4:**
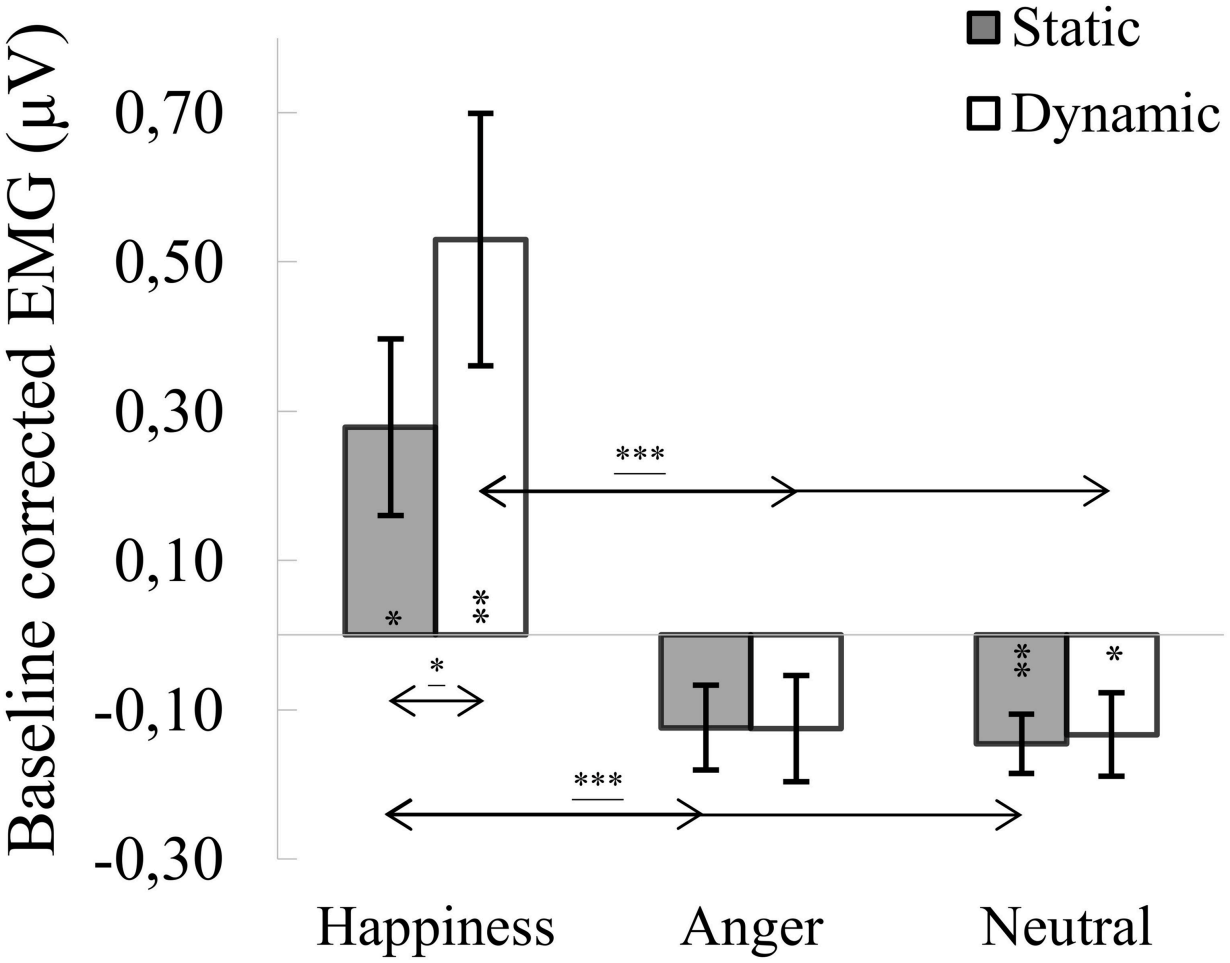
Mean (±SE) EMG activity changes and corresponding statistics for zygomaticus major during presentation conditions. Asterisks with lines beneath indicate significant differences between conditions (simple effects) in EMG responses: ^*^p < 0.05, ^***^p < 0.001. Separate asterisks indicate significant differences from baseline EMG responses: ^*^p < 0.05, ^**^p < 0.01.

ANOVA showed a significant main effect of expression [*F*_(1.142, 43.404)_ = 11.060, *p* < 0.001, η2 = 0.225], indicating that activity of the ZM for happiness [*M* = 0.404, *SE* = 0.138] was increased compared to angry [*M* = −0.125, *SE* = 0.054; *t*_(36)_ = 3.458, *p* = 0.004] and neutral expressions [*M* = −0.140, *SE* = 0,043; *t*_(36)_ = 3.358, *p* = 0.005]. The main effect of modality approached significance [*F*_(1, 38)_ = 3.545, *p* = 0.067, η2 = 0.085], with activity of the ZM greater for dynamic [*M* = 0.091, *SE* = 0.091] than static [*M* = 0.003, *SE* = 0.043] facial expressions. A significant expression × modality interaction [*F*_(1.788, 67.943)_ = 4.385, *p* = 0.020, η2 = 0.103] revealed that ZM activity was higher for dynamic than for static happiness [*t*_(33)_ = 2.681, *p* = 0.011]. Higher ZM activity was observed in dynamic happiness compared to angry [*t*_(33)_ = 3.541, *p* = 0.003] and neutral [*t*_(33)_ = 3.354, *p* = 0.006] facial expressions. Results of higher ZM activity were observed during comparison of static happiness with static angry [*t*_(33)_ = 3.124, *p* = 0.011] and neutral [*t*_(33)_ = 3.050, *p* = 0.013] facial expression conditions (see Figure 4).

One-sample *t*-tests revealed increased ZM activity, compared to baseline, for dynamic [*t*_(40)_ = 3.217, *p* = 0.003] and static [*t*_(40)_ = 2.415, *p* = 0.020] happiness and lower activity for dynamic [*t*_(39)_ = −2.307, *p* = 0.026] and static [*t*_(39)_ = 3.612, *p* = 0.001] neutral facial expressions. Mean ZM activity for anger did not differ from baseline [*t*_(39) dynamic_ = −0.688, *p* = 0.498; *t*_(41) static_ = −1.589, *p* = 0.120].

### fMRI data

Region of interest (ROI) analyses were carried out for the contrasts comparing brain activation during dynamic vs. static expressions, resulting in 11 contrasts of interest: happiness dynamic > happiness static, anger dynamic > anger static, neutral dynamic > neutral static, emotion dynamic > emotion static (emotion dynamic—pooled dynamic happiness, and anger conditions; emotion static—similar pooling), all dynamic > all static (all dynamic—pooled dynamic happiness, anger and neutral conditions; all static—similar pooling), happiness dynamic > neutral dynamic, happiness static > neutral static, anger dynamic > neutral dynamic, anger static > neutral static, emotion dynamic > neutral dynamic, emotion static > neutral static. Mentioned contrasts were calculated in order to investigate two types of questions. The contrast emotion/happiness/anger/all dynamic/static > neutral dynamic/static addresses neural correlates of FM of emotional/happiness/anger/all expressions. The other contrasts (i.e., emotion/happiness/anger/all dynamic > emotion/happiness/anger/all static) relate to the difference in processing between dynamic and static stimuli.

ROI analyses indicated that for the happiness dynamic > happiness static contrast, V5/MT+ and STS were activated bilaterally. Other structures for the contrast were activated only in the right hemisphere (i.e., pre-SMA, IPL, BA45) (see Table 1; for whole brain analysis see Supplementary Table 1).

**Table 1:** Summary statistics of ROIs' activations for happiness dynamic > happiness static contrast.

**Region of interest**	**Left hemisphere**			**Right hemisphere**		
	***M***	***t***	***p***	***M***	***t***	***p***
V5/MT+	0.466	9.876	0.000^***^	0.877	12.049	0.000^***^
Primary Motor Cortex	−0.023	−0.533	0.702	−0.031	−0.653	0.741
Premotor Cortex	0.007	0.217	0.415	0.036	1.145	0.129
Pre-SMA	0.020	0.532	0.299	0.160	3.807	0.000^**^
Primary Somatosensory Cortex	0.006	0.148	0.441	0.045	0.986	0.165
Inferior Parietal Lobule	0.068	2.007	0.025	0.156	3.698	0.000^**^
Superior Temporal Sulcus	0.298	10.031	0.000^***^	0.441	11.778	0.000^***^
BA44	0.097	2.441	0.009	0.076	2.413	0.010
BA45	0.093	2.105	0.020	0.140	4.678	0.000^***^
Amygdala	0.080	2.136	0.019	0.088	2.516	0.008
Anterior Cingulate Cortex	0.004	0.140	0.445	0.013	0.564	0.288
Insula	0.056	2.379	0.011	0.044	1.766	0.042
Caudate Head	0.071	1.757	0.043	0.090	2.166	0.018
Putamen	0.053	2.046	0.023	0.060	2.428	0.010
Nucleus Accumbens	0.033	0.879	0.192	0.061	1.745	0.044
Globus Pallidus	0.049	2.254	0.015	0.050	2.766	0.004

Asterisks indicate significant, Bonferroni corrected, activations of each ROI:

** *p < 0.01*,

*** *p < 0.001*.

For the anger dynamic > anger static contrast, V5/MT+ and STS were also activated bilaterally. However, this contrast revealed additional bilateral activation of the amygdala. Other structures revealed by this contrast were visible only in the right hemisphere (i.e., pre-SMA and BA45) (see Table 2; for whole brain analysis see Supplementary Table 2).

**Table 2:** Summary statistics of ROIs' activations for anger dynamic > anger static contrast.

**Region of interest**	**Left hemisphere**			**Right hemisphere**		
	***M***	***t***	***p***	***M***	***t***	***p***
V5/MT+	0.475	9.499	0.000^***^	1.042	12.104	0.000^***^
Primary Motor Cortex	0.025	0.603	0.275	0.032	0.742	0.231
Premotor Cortex	0.013	0.386	0.351	0.057	1.733	0.045
Pre-SMA	0.032	0.846	0.201	0.163	4.087	0.000^**^
Primary Somatosensory Cortex	0.026	0.610	0.272	0.071	1.579	0.061
Inferior Parietal Lobule	0.029	0.798	0.214	0.065	1.531	0.066
Superior Temporal Sulcus	0.290	9.373	0.000^***^	0.450	11.635	0.000^***^
BA44	0.053	1.261	0.107	0.057	1.762	0.042
BA45	0.108	2.197	0.017	0.134	3.834	0.000^**^
Amygdala	0.130	3.736	0.000^**^	0.151	4.606	0.000^***^
Anterior Cingulate Cortex	−0.031	−1.000	0.839	−0.021	−0.649	0.740
Insula	0.015	0.526	0.301	0.036	1.345	0.093
Caudate Head	0.016	0.414	0.340	0.058	1.423	0.081
Putamen	0.033	1.138	0.131	0.057	2.219	0.016
Nucleus Accumbens	0.014	0.379	0.353	0.024	0.612	0.272
Globus Pallidus	0.030	1.148	0.128	0.021	1.070	0.145

Asterisks indicate significant, Bonferroni corrected, activations of each ROI:

** *p < 0.01*,

*** *p < 0.001*.

For the neutral dynamic > neutral static contrast, only V5/MT+ and STS were activated bilaterally (see Table 3; for whole brain analysis see Supplementary Tables 3–5).

**Table 3:** Summary statistics of ROIs' activations for neutral dynamic > neutral static contrast.

**Region of interest**	**Left hemisphere**			**Right hemisphere**		
	***M***	***t***	***p***	***M***	***t***	***p***
V5/MT+	0.220	5.029	0.000^***^	0.548	13.479	0.000^***^
Primary Motor Cortex	0.020	0.680	0.250	0.018	0.623	0.268
Premotor Cortex	0.015	0.560	0.289	0.042	1.783	0.041
Pre-SMA	−0.003	−0.051	0.520	0.082	1.698	0.048
Primary Somatosensory Cortex	0.018	0.711	0.240	0.030	1.048	0.150
Inferior Parietal Lobule	−0.006	−0.196	0.577	0.050	1.605	0.058
Superior Temporal Sulcus	0.155	5.567	0.000^***^	0.241	7.330	0.000^***^
BA44	0.058	1.977	0.027	0.055	2.110	0.020
BA45	0.008	0.192	0.424	0.075	2.675	0.005
Amygdala	0.034	1.138	0.130	0.049	1.939	0.029
Anterior Cingulate Cortex	−0.030	−0.995	0.837	−0.016	−0.618	0.730
Insula	0.021	1.087	0.141	0.026	1.459	0.076
Caudate Head	0.027	0.671	0.253	0.037	1.068	0.146
Putamen	0.020	0.733	0.234	0.021	0.937	0.177
Nucleus Accumbens	0.018	0.484	0.315	0.039	1.077	0.144
Globus Pallidus	0.006	0.257	0.399	0.033	1.553	0.064

Asterisks indicate significant, Bonferroni corrected, activations of each ROI:

*** *p < 0.001*.

The emotion dynamic > emotion static contrast, revealed bilateral activations of V5/MT+, STS, BA45, BA44, and Amygdala. Additionally, this contrast revealed activations of Putamen, Globus Pallidus, IPL and pre-SMA in the right hemisphere (see Table 4; for whole brain analysis see Supplementary Table 4).

**Table 4:** Summary statistics of ROIs' activations for emotion dynamic > emotion static contrast.

**Region of interest**	**Left hemisphere**			**Right hemisphere**		
	***M***	***t***	***p***	***M***	***t***	***p***
V5/MT+	0.941	11.335	0.000^***^	1.920	13.702	0.000^***^
Primary Motor Cortex	0.002	0.028	0.489	0.001	0.018	0.493
Premotor Cortex	0.019	0.426	0.336	0.094	2.038	0.024
pre-SMA	0.052	0.978	0.167	0.323	5.353	0.000^***^
Primary Somatosensory Cortex	0.033	0.505	0.308	0.116	1.710	0.047
Inferior Parietal Lobule	0.097	2.186	0.017	0.221	3.638	0.000^*^
Superior Temporal Sulcus	0.588	12.633	0.000^***^	0.892	16.607	0.000^***^
BA44	0.150	3.026	0.002^+^	0.133	3.024	0.002^+^
BA45	0.201	3.683	0.000^**^	0.274	6.151	0.000^***^
Amygdala	0.211	4.351	0.000^**^	0.239	5.485	0.000^***^
Anterior Cingulate Cortex	−0.027	−0.680	0.750	−0.007	−0.172	0.568
Insula	0.070	1.797	0.040	0.080	2.062	0.022
Caudate Head	0.088	1.698	0.048	0.148	2.475	0.009
Putamen	0.086	2.352	0.012	0.117	3.676	0.000^*^
Nucleus Accumbens	0.048	0.973	0.168	0.086	1.714	0.047
Globus Pallidus	0.079	2.510	0.008	0.071	2.985	0.002^+^

Asterisks indicate significant, Bonferroni corrected, activations of each ROI:

+ *p < 0.1*,

* *p < 0.05*,

** *p < 0.01*,

*** *p < 0.001*.

The all dynamic > all static contrast, illustrating general processing of dynamic compared to static expressions, revealed bilateral activations of V5/MT+, STS, BA44, BA45, and the amygdala. Moreover, few cortical areas and subcortical structures were activated only in the right hemisphere (i.e., premotor cortex (trend effect), pre-SMA, IPL, caudate head, putamen and globus pallidus) (see Table 5; for whole brain analysis see Supplementary Table 5).

**Table 5:** Summary statistics of ROIs' activations for all dynamic > all static expressions contrast.

**Region of interest**	**Left hemisphere**			**Right hemisphere**		
	***M***	***t***	***p***	***M***	***t***	***p***
V5/MT+	1.161	11.284	0.000^***^	2.468	14.916	0.000^***^
Primary Motor Cortex	0.021	0.332	0.371	0.020	0.298	0.384
Premotor Cortex	0.034	0.697	0.245	0.135	2.884	0.003^+^
pre-SMA	0.050	0.799	0.214	0.405	5.733	0.000^***^
Primary Somatosensory Cortex	0.051	0.759	0.226	0.146	1.949	0.029
Inferior Parietal Lobule	0.091	1.866	0.034	0.271	3.974	0.000^**^
Superior Temporal Sulcus	0.743	12.457	0.000^***^	1.132	15.947	0.000^***^
BA44	0.208	3.914	0.000^**^	0.187	4.035	0.000^**^
BA45	0.209	3.851	0.000^**^	0.349	7.289	0.000^***^
Amygdala	0.245	4.192	0.000^**^	0.288	5.572	0.000^***^
Anterior Cingulate Cortex	−0.057	−1.235	0.888	−0.024	−0.505	0.692
Insula	0.091	2.113	0.020	0.105	2.427	0.010
Caudate Head	0.114	2.088	0.021	0.185	3.209	0.001^*^
Putamen	0.107	2.752	0.004	0.137	4.119	0.000^**^
Nucleus Accumbens	0.065	1.208	0.117	0.124	2.186	0.017
Globus Pallidus	0.085	2.687	0.005	0.104	4.116	0.000^**^

Asterisks indicate significant, Bonferroni corrected, activations of each ROI:

+ *p < 0.1*,

* *p < 0.05*,

** *p < 0.01*,

*** *p < 0.001*.

The happiness dynamic > neutral dynamic contrast, showed bilateral activations of V5/MT+, STS, pre-SMA, IPL, BA45, Amygdala, Anterior Cingulate Cortex, Caudate Head, Putamen, and Globus Pallidus. Activation of BA44 was visible only in the left hemisphere (see Table 6; for whole brain analysis see Supplementary Table 6).

**Table 6:** Summary statistics of ROIs' activations for happiness dynamic > neutral dynamic contrast.

**Region of interest**	**Left hemisphere**			**Right hemisphere**		
	***M***	***t***	***p***	***M***	***t***	***p***
V5/MT+	0.287	6.446	0.000^***^	0.362	7.390	0.000^***^
Primary Motor Cortex	−0.015	−0.383	0.648	−0.016	−0.366	0.642
Premotor Cortex	0.029	0.918	0.182	0.031	1.031	0.154
pre-SMA	0.172	3.292	0.001^*^	0.231	4.697	0.000^***^
Primary Somatosensory Cortex	0.033	0.852	0.199	0.069	1.804	0.039
Inferior Parietal Lobule	0.151	4.234	0.000^**^	0.121	2.913	0.003^+^
Superior Temporal Sulcus	0.184	6.481	0.000^***^	0.212	6.974	0.000^***^
BA44	0.111	3.159	0.001^*^	0.073	2.473	0.009
BA45	0.177	3.858	0.000^**^	0.124	3.562	0.000^*^
Amygdala	0.140	4.806	0.000^***^	0.097	3.500	0.001^*^
Anterior Cingulate Cortex	0.109	3.708	0.000^**^	0.086	3.421	0.001^*^
Insula	0.022	0.986	0.165	0.017	0.772	0.222
Caudate Head	0.120	2.954	0.002^+^	0.153	3.826	0.000^**^
Putamen	0.074	2.979	0.002^+^	0.063	2.885	0.003^+^
Nucleus Accumbens	0.074	2.074	0.022	0.083	2.385	0.011
Globus Pallidus	0.089	3.983	0.000^**^	0.073	3.703	0.000^**^

Asterisks indicate significant, Bonferroni corrected, activations of each ROI:

+ *p < 0.1*,

* *p < 0.05*,

** *p < 0.01*,

*** *p < 0.001*.

For the happiness static > neutral static contrast, only pre-SMA was activated bilaterally (see Table 7; for whole brain analysis see Supplementary Table 7).

**Table 7:** Summary statistics of ROIs' activations for happiness static > neutral static contrast.

**Region of interest**	**Left hemisphere**			**Right hemisphere**		
	***M***	***t***	***p***	***M***	***t***	***p***
V5/MT+	0.041	1.098	0.139	0.032	0.850	0.200
Primary Motor Cortex	0.028	0.737	0.233	0.033	0.873	0.194
Premotor Cortex	0.037	1.340	0.093	0.036	1.341	0.093
pre-SMA	0.150	3.794	0.000^**^	0.152	3.962	0.000^**^
Primary Somatosensory Cortex	0.045	1.278	0.104	0.054	1.503	0.070
Inferior Parietal Lobule	0.078	2.657	0.005	0.014	0.483	0.316
Superior Temporal Sulcus	0.041	1.544	0.065	0.012	0.413	0.341
BA44	0.072	1.800	0.039	0.052	1.863	0.035
BA45	0.092	2.058	0.023	0.059	2.035	0.024
Amygdala	0.094	2.759	0.004	0.058	2.062	0.023
Anterior Cingulate Cortex	0.075	2.843	0.003	0.056	2.326	0.012
Insula	−0.013	−0.554	0.709	−0.002	−0.069	0.527
Caudate Head	0.076	1.842	0.036	0.100	2.651	0.006
Putamen	0.041	1.357	0.091	0.024	0.850	0.200
Nucleus Accumbens	0.059	1.541	0.065	0.061	1.593	0.059
Globus Pallidus	0.046	1.998	0.026	0.056	2.833	0.003

Asterisks indicate significant, Bonferroni corrected, activations of each ROI:

** *p < 0.01*.

For the anger dynamic > neutral dynamic contrast, analysis revealed bilateral activations of V5/MT+, STS, Amygdala and BA45. Pre-SMA activation was visible only in the right hemisphere (see Table 8; for whole brain analysis see Supplementary Table 8).

**Table 8:** Summary statistics of ROIs' activations for anger dynamic > neutral dynamic contrast.

**Region of interest**	**Left hemisphere**			**Right hemisphere**		
	***M***	***t***	***p***	***M***	***t***	***p***
V5/MT+	0.320	8.242	0.000^***^	0.567	9.956	0.000^***^
Primary Motor Cortex	−0.023	−0.689	0.753	0.002	0.067	0.474
Premotor Cortex	0.005	0.156	0.438	0.022	0.801	0.214
pre-SMA	0.142	2.742	0.004	0.211	4.613	0.000^***^
Primary Somatosensory Cortex	0.010	0.342	0.367	0.050	1.538	0.065
Inferior Parietal Lobule	0.080	2.110	0.020	0.028	0.735	0.233
Superior Temporal Sulcus	0.216	7.995	0.000^***^	0.281	8.845	0.000^***^
BA44	0.074	1.876	0.034	0.043	1.492	0.071
BA45	0.166	3.059	0.002^+^	0.108	3.228	0.001^*^
Amygdala	0.119	3.942	0.000^**^	0.116	4.043	0.000^**^
Anterior Cingulate Cortex	0.016	0.631	0.266	0.008	0.308	0.380
Insula	0.011	0.519	0.303	0.013	0.799	0.214
Caudate Head	0.029	0.725	0.236	0.076	2.001	0.026
Putamen	0.039	1.469	0.074	0.044	1.914	0.031
Nucleus Accumbens	0.043	1.178	0.122	0.045	1.429	0.080
Globus Pallidus	0.056	2.221	0.016	0.038	1.842	0.036

Asterisks indicate significant, Bonferroni corrected, activations of each ROI:

+ *p < 0.1*,

* *p < 0.05*,

** *p < 0.01*,

*** *p < 0.001*.

The anger static > neutral static contrast, revealed no significant activations of brain structures (see Table 9; for whole brain analysis see Supplementary Table 9).

**Table 9:** Summary statistics of ROIs' activations for anger static > neutral static contrast.

**Region of interest**	**Left hemisphere**			**Right hemisphere**		
	***M***	***t***	***p***	***M***	***t***	***p***
V5/MT+	0.064	1.551	0.064	0.073	1.576	0.061
Primary Motor Cortex	−0.029	−0.742	0.769	−0.012	−0.282	0.610
Premotor Cortex	0.007	0.221	0.413	0.006	0.179	0.429
pre-SMA	0.108	2.227	0.016	0.131	2.388	0.011
Primary Somatosensory Cortex	0.002	0.063	0.475	0.008	0.243	0.404
Inferior Parietal Lobule	0.045	1.176	0.123	0.013	0.346	0.366
Superior Temporal Sulcus	0.081	2.570	0.007	0.071	1.950	0.029
BA44	0.080	2.130	0.019	0.041	1.175	0.123
BA45	0.065	1.383	0.087	0.048	1.337	0.094
Amygdala	0.023	0.799	0.214	0.014	0.537	0.297
Anterior Cingulate Cortex	0.016	0.591	0.279	0.012	0.433	0.334
Insula	0.017	0.643	0.262	0.003	0.125	0.450
Caudate Head	0.039	0.982	0.166	0.055	1.353	0.091
Putamen	0.027	0.952	0.173	0.009	0.349	0.365
Nucleus Accumbens	0.046	1.185	0.121	0.060	1.578	0.061
Globus Pallidus	0.033	1.414	0.082	0.049	2.023	0.025

The emotion dynamic > neutral dynamic contrast, showed that V5/MT+, STS, Amygdala, BA45, pre-SMA and Globus Pallidus were activated bilaterally. Additionally, this contrast revealed IPL activation in the left hemisphere and Caudate Head activation in the right hemisphere (see Table 10; for whole brain analysis see Supplementary Table 10).

**Table 10:** Summary statistics of ROIs' activations for emotion dynamic > neutral dynamic contrast.

**Region of interest**	**Left hemisphere**			**Right hemisphere**		
	***M***	***t***	***p***	***M***	***t***	***p***
V5/MT+	0.608	7.841	0.000^***^	0.929	10.022	0.000^***^
Primary Motor Cortex	−0.039	−0.642	0.738	−0.014	−0.216	0.585
Premotor Cortex	0.034	0.637	0.264	0.053	1.091	0.141
pre-SMA	0.315	3.189	0.001^*^	0.442	5.096	0.000^***^
Primary Somatosensory Cortex	0.043	0.791	0.217	0.118	2.032	0.024
Inferior Parietal Lobule	0.232	3.626	0.000^*^	0.149	2.159	0.018
Superior Temporal Sulcus	0.400	8.073	0.000^***^	0.493	9.238	0.000^***^
BA44	0.185	3.149	0.001^*^	0.117	2.528	0.008
BA45	0.343	3.908	0.000^**^	0.233	3.867	0.000^**^
Amygdala	0.259	5.110	0.000^***^	0.213	4.497	0.000^***^
Anterior Cingulate Cortex	0.125	2.626	0.006	0.094	2.206	0.016
Insula	0.033	0.930	0.179	0.030	0.986	0.165
Caudate Head	0.149	2.041	0.024	0.230	3.247	0.001^*^
Putamen	0.113	2.514	0.008	0.108	2.809	0.004
Nucleus Accumbens	0.117	1.869	0.034	0.129	2.241	0.015
Globus Pallidus	0.146	3.364	0.001^*^	0.111	3.077	0.002^+^

Asterisks indicate significant, Bonferroni corrected, activations of each ROI:

+ *p < 0.1*,

* *p < 0.05*,

** *p < 0.01*,

*** *p < 0.001*.

For the emotion static > neutral static contrast, ROI analysis revealed only pre-SMA activations (see Table 11; for whole brain analysis see Supplementary Table 11).

**Table 11:** Summary statistics of ROIs' activations for emotion static > neutral static contrast.

**Region of interest**	**Left hemisphere**			**Right hemisphere**		
	***M***	***t***	***p***	***M***	***t***	***p***
V5/MT+	0.106	1.511	0.069	0.105	1.456	0.076
Primary Motor Cortex	−0.002	−0.024	0.509	0.022	0.316	0.377
Premotor Cortex	0.044	0.856	0.198	0.042	0.805	0.212
pre-SMA	0.258	3.331	0.001^*^	0.283	3.411	0.001^*^
Primary Somatosensory Cortex	0.047	0.720	0.238	0.062	1.045	0.151
Inferior Parietal Lobule	0.123	2.228	0.015	0.028	0.521	0.302
Superior Temporal Sulcus	0.122	2.403	0.010	0.083	1.495	0.071
BA44	0.152	2.244	0.015	0.093	1.702	0.048
BA45	0.158	2.020	0.025	0.108	1.977	0.027
Amygdala	0.117	2.304	0.013	0.072	1.658	0.052
Anterior Cingulate Cortex	0.092	1.905	0.032	0.068	1.490	0.072
Insula	0.004	0.098	0.461	0.002	0.041	0.484
Caudate Head	0.115	1.666	0.051	0.155	2.307	0.013
Putamen	0.068	1.313	0.098	0.032	0.731	0.234
Nucleus Accumbens	0.105	1.556	0.063	0.120	1.809	0.039
Globus Pallidus	0.080	1.966	0.028	0.105	2.722	0.005

Asterisks indicate significant, Bonferroni corrected, activations of each ROI:

* *p < 0.05*.

### Correlation analysis

#### Muscle-brain correlations of dynamic and static happiness conditions

Correlation analyses computed for the happiness dynamic condition with ZM revealed positive relations bilaterally in the pre-SMA (trend effect), putamen, nucleus accumbens and globus pallidus. Trend effects were found in the activations of the right BA44 and insular cortex. No relationships were found between brain activity in the happiness dynamic conditions and OO muscle activity. For the CS, negative relations were found for V5/MT+, STS, BA45 in the left hemisphere, while IPL and ACC in the right hemisphere. Negative trend relationships were found bilaterally in the caudate head (see Table 12).

**Table 12:** Muscles-brain correlations of dynamic and static happiness conditions.

**Region of interest**	**Happiness dynamic**						**Happiness static**					
	**CS**		**ZM**		**OO**		**CS**		**ZM**		**OO**	
	**LH**	**RH**	**LH**	**RH**	**LH**	**RH**	**LH**	**RH**	**LH**	**RH**	**LH**	**RH**
V5/MT+	−0.36^*^	−0.111	0.200	0.077	0.077	0.086	−0.045	0.078	0.005	0.001	−0.062	−0.120
Primary Motor Cortex	0.036	0.038	−0.085	−0.030	−0.126	−0.130	−0.292^+^	−0.283^+^	0.096	0.052	0.301^+^^b^	0.312^*^^b^
Premotor Cortex	−0.002	−0.046	0.140	0.246	−0.081	0.071	−0.362^*^	−0.285^+^	0.206	0.106	0.275^+^	0.223
pre-SMA	−0.233	−0.256	0.267^+^	0.297^+^^b^	0.104	0.171	−0.081	−0.078	0.144	0.108	−0.008	−0.031
Primary Somatosensory Cortex	−0.124	−0.163	0.078	0.111	−0.024	−0.083	−0.317^*^	−0.309^*^	0.013	0.100	0.257	0.400^*^^b^
Inferior Parietal Lobule	−0.236	−0.39^*^	0.130	0.210	−0.106	0.037	−0.149	−0.139	−0.025	−0.113	0.083	0.017
Superior Temporal Sulcus	−0.334^*^	−0.232	0.193	0.323^*^^b^	0.095	0.196	−0.191	−0.097	0.150	0.000	0.124	0.035
BA44	−0.188	−0.218	0.183	0.293^+^	−0.013	0.033	−0.377^*^	−0.250	0.244	−0.041	0.166	0.059
BA45	−0.352^*^	−0.169	0.035	0.250	−0.061	0.024	−0.111	−0.044	0.080	−0.109	−0.038	−0.034
Amygdala	−0.232	−0.108	0.232	0.140	0.029	−0.122	−0.238	−0.232	0.129	0.161	0.110	0.120
Anterior Cingulate Cortex	−0.255	−0.326^*^	0.089	0.097	0.086	0.114	−0.103	−0.097	−0.038	0.015	0.001	−0.009
Insula	−0.135	−0.191	0.140	0.286^+^	−0.077	0.018	−0.227	−0.205	0.418^**^^b^	0.276^+^^b^	0.333^*^	0.265^+^
Caudate Head	−0.280^+^	−0.300^+^	0.237	0.355^*^	0.131	0.149	−0.253	−0.141	0.201	0.149	0.266^+^	0.213
Putamen	−0.124	−0.141	0.352^*^	0.458^**^^b^	0.021	0.112	−0.181	−0.099	0.397^*^^b^	0.294^+^^b^	0.201	0.037
Nucleus Accumbens	−0.187	−0.221	0.314^*^^b^	0.357^*^^b^	0.031	0.158	−0.056	−0.098	0.150	0.013	−0.005	−0.138
Globus Pallidus	−0.209	−0.224	0.418^**^^b^	0.411^**^^b^	0.151	0.117	−0.142	−0.146	0.338^*^^b^	0.202	0.164	0.090

Post-number asterisks indicate significant Pearson correlations of muscle-ROI pairs:

+ *p < 0.1*,

* *p < 0.05*,

** *p < 0.01*.

*Addition “b” letter indicates that Pearson coefficient is significant based on BCa bootstrap criterion. CS, corrugator supercilii; ZM, zygomaticus major; OO, orbicularis oculi, LH, left hemisphere; RH, right hemisphere*.

Correlation analyses computed for the happiness static condition with ZM indicated positive relationships for the left insula, putamen and globus pallidus. Trend positive effects of ZM and brain activity were found in the right insula and putamen. Positive relationships of the OO and brain activity during perception of the happiness static condition were found in the right primary motor cortex, right primary somatosensory cortex and left insula. Trend effects of the OO and brain activity were observed for the left primary motor cortex, premotor cortex and caudate head. Negative relationships of the CS and brain activity were found for the premotor cortex and BA44 in the left hemisphere. Moreover, CS activity was negatively related to activity of the primary somatosensory cortex (bilaterally), primary motor cortex (bilaterally) and premotor cortex (right) (see Table 12).

#### Muscle-brain correlations of dynamic and static anger conditions

Correlation analyses performed for the anger dynamic condition indicated a negative relationship of the CS and activity in left BA44. The positive relationship was found with the OO and brain activity during perception of dynamic angry expressions in the STS (bilaterally) and right premotor cortex. Trend positive relationships were found in the primary motor cortex (bilaterally) and right BA45, amygdala and insula (see Table 13).

**Table 13:** Muscles-brain correlations of dynamic and static anger conditions.

**Region of interest**	**Anger dynamic**				**Anger static**			
	**CS**		**OO**		**CS**		**OO**	
	**LH**	**RH**	**LH**	**RH**	**LH**	**RH**	**LH**	**RH**
V5/MT+	0.082	−0.089	0.159	0.188	0.178	0.068	−0.287^+^	−0.117
Primary Motor Cortex	−0.123	−0.022	0.292^+^^b^	0.289^+^	0.001	0.044	0.131	0.113
Premotor Cortex	−0.252	−0.101	0.251	0.316^*^^b^	−0.079	−0.017	0.257	0.238
pre-SMA	−0.047	−0.077	0.201	0.026	−0.148	−0.084	0.312^*^	0.35^*^
Primary Somatosensory Cortex	−0.190	−0.085	0.096	0.162	0.019	0.119	0.149	0.051
Inferior Parietal Lobule	−0.116	−0.003	0.109	0.241	0.240	0.276^+^^b^	0.094	0.106
Superior Temporal Sulcus	0.103	0.093	0.319^*^^b^	0.369^*^^b^	0.174	0.305^*^	−0.004	0.095
BA44	−0.323^*^	−0.069	−0.067	0.124	0.022	0.042	0.207	0.174
BA45	−0.221	−0.157	0.195	0.288^+^^b^	0.050	0.053	0.273^+^	0.186
Amygdala	−0.114	−0.262	0.189	0.284^+^	−0.034	0.024	0.108	−0.137
Anterior Cingulate Cortex	0.021	−0.058	0.089	0.036	0.027	0.098	0.159	0.139
Insula	−0.006	0.134	0.145	0.275^+^^b^	0.094	0.183	0.148	0.091
Caudate Head	−0.014	0.015	0.049	0.136	0.026	0.016	0.233	0.276^+^
Putamen	−0.081	−0.012	0.135	0.159	−0.004	0.052	−0.053	−0.128
Nucleus Accumbens	−0.198	−0.091	−0.011	0.182	−0.030	0.097	0.189	0.084
Globus Pallidus	−0.174	−0.022	0.159	0.196	0.046	0.048	−0.007	0.120

Post-number asterisks indicate significant Pearson correlations of muscle-ROI pairs:

+ *p < 0.1*,

* p < 0.05.

*Addition “b” letter indicates that Pearson coefficient is significant based on BCa bootstrap criterion. LH, left hemisphere; RH, right hemisphere*.

Positive relationships of brain and CS activity for static anger were observed in the right STS and right IPL (trend effect). Activity in the pre-SMA (bilaterally) was positively related to OO activity during perception of angry pictures. Trend effects of the relationship between the OO and brain activity during perception of angry static conditions were observed in the right caudate (positive), left BA45 (positive) and in V5/MT+ (negative, see Table 13).

## Discussion

The present study examined neural correlates of FM during the observation of dynamic compared to static facial expressions. Proofs of concept came from facial EMG, fMRI, and combined EMG-fMRI analyses. Firstly, the anticipated patterns of mimicry were observed, demonstrated by increased ZM and OO activity and decreased CS activity for happiness (Rymarczyk et al., 2016), as well as increased CS activity for anger (Dimberg and Petterson, 2000). Moreover, we found that dynamic presentations of happy facial expressions induced higher EMG amplitude in the ZM, OO, and CS compared to static presentations. Angry facial expression were not associated with differences in the CS response between static and dynamic displays. Analysis of fMRI data revealed that dynamic (compared to static) emotional expressions activated bilateral STS, V5/MT+, and frontal and parietal areas. On the other hand, the perception of neutral dynamic compared to neutral static facial displays activated only structures related to biological motion i.e., bilaterally V5/MT+ and STS. Furthermore, some interaction effects of emotion and modality were found. For example, dynamic compared to static displays induced greater activity in the bilateral amygdala for anger, while this effect was found in the right IPL for happiness. The correlations between brain activity and facial muscle reactions revealed that correlated regions are related to the motor simulation of facial expressions, such as the IFG, which is considered a classical MNS. Conversely, the correlations between brain activity and facial muscle reactions also demonstrate a role in emotional processing, such as in the insula, which is part of extended MNS.

### EMG response for dynamic compared to static facial expressions

The recorded EMG data showed that the subjects reacted spontaneously to happy facial expressions with increased ZM and OO activity (Rymarczyk et al., 2016) and decreased CS activity, interpretable as FM (Dimberg and Thunberg, 1998). However, EMG responses observed in our study were low in amplitude but comparable to other reports (Sato et al., 2008; Dimberg et al., 2011; Rymarczyk et al., 2011). In all muscles, the response was more pronounced when dynamic happy stimuli were presented (Weyers et al., 2006; Sato et al., 2008; Rymarczyk et al., 2011), which points to the benefits of applying dynamic stimuli (Murata et al., 2016). Patterns of ZM and OO reactions observed for dynamic happiness could be interpreted as a Duchenne smile (Ekman et al., 1990), suggesting that subjects could experience true and genuine positive emotion. Moreover, we observed higher CS reactions, similar for static and dynamic anger conditions, showing typical evidence of FM for this emotion (Sato et al., 2008; Dimberg et al., 2011). Increased CS response was found for neutral facial expressions as well. Some studies have reported increased CS activity as a function of mental effort (Neumann and Strack, 2000), disapproval (Cannon et al., 2011) or global negative affect (Larsen et al., 2003). In the case of our study, we interpret that increased CS activity for neutral facial expressions was a consequence of the instruction used in the procedure that asked subjects to pay careful attention (i.e., mental effort) to observed actors.

### Neural network for dynamic compared to static facial expressions

We found that passive viewing of emotional dynamic, compared to neutral dynamic stimuli, activated a wide network of brain regions. This network included the inferior frontal gyrus (left BA44 and bilaterally BA45), left IPL, bilaterally preSMA, STS and V5/MT+, as well as left and right amygdala, right caudate head and bilaterally pallidus. In contrast, the emotional static displays compared to neutral displays activated only bilateral preSMA. The last mentioned neuronal pattern was significant due to happiness. Furthermore, dynamic happiness evoked activity that was greater than static happiness in the right IPL, while dynamic vs. static angry faces evoked greater bilateral activity in the amygdala. As expected, we found that, regardless of a specific emotion, dynamic stimuli selectively activated the bilateral visual area V5/MT+ and superior temporal sulcus, structures associated with motion and biological motion perception, respectively (Robins et al., 2009; Arsalidou et al., 2011; Foley et al., 2012; Furl et al., 2015). Recently, in a magneto-encephalography (MEG) study, Sato et al. (2015) explored temporal profiles and dynamic interaction patterns of brain activity during perception of dynamic emotional facial expressions in comparison to dynamic mosaics. Notably, they found that, apart from V5/MT+ and STS, the right IFG exhibited higher activity for dynamic faces vs. dynamic mosaics. Furthermore, they have found a direct functional connectivity between the STS and IFG, closely related to FM.

Our findings concerning IFG are in line with those of previous studies (Carr et al., 2003; Leslie et al., 2004) suggesting that perception of emotional facial displays involves a classical MNS, which is sensitive to goal-directed actions. There is an assumption that during the observation of another individual's actions, the brain simulates the same action by activating the neurons localized in the IFG, which are involved in executing the same behavior (Jabbi and Keysers, 2008). For example, Carr et al. (2003) asked subjects to observe and imitate static emotional facial expressions and found that both tasks induced extensive activity in the IFG. Activation of the right IFG and parietal cortex were also found during passive viewing of dynamic compared to static emotional facial expressions (Arsalidou et al., 2011; Foley et al., 2012) or during viewing and executing smiles (Hennenlotter et al., 2005). It was suggested that activated mirror neurons localized in the IFG and parietal regions could convert observed emotional facial expressions into a pattern of neural activity that would be suitable for producing similar facial expressions, and would provide the basis for a motor simulation of facial expressions (Gazzola et al., 2006; van der Gaag et al., 2007; Jabbi and Keysers, 2008). Our results seems to be in line with the perception-behavior link model (Chartrand and Bargh, 1999), which assumes that an observer's motor system “resonates” and facilitates the understanding of the perceived action. It is believed that classical MNS are responsible for such processes (for a review, see Bastiaansen et al., 2009). Moreover, it seems that dynamic emotional facial expressions might be a stronger social signal to induce imitation processes in MNS, since we did not observe activity of IFG when comparing neutral dynamic to neutral static facial expressions' contrasts.

To summarize, our findings have revealed that functional properties of classical MNS manifest mainly during perception of dynamic compared to static facial displays. It may be justified that dynamic stimuli, which are relevant for social interaction, engage a wide network of brain regions sensitive to motion stimuli (Kilts et al., 2003; Kessler et al., 2011) and signaling intentions (Gallagher et al., 2000; Pelphrey et al., 2003), and thus may be a strong social signal to induce simulation processes in MNS.

### Relationships between neural activity and facial muscle responses

One of the fundamental questions regarding the neural basis of FM is whether this phenomenon involves motor and/or affective representations of observed expressions (for a review, see Bastiaansen et al., 2009). So far, only one study has examined that question with simultaneous recording of EMG and BOLD signal, However, only static avatar emotional expressions were used (Likowski et al., 2012). In our study, where both static and dynamic natural displays were applied, the associations between activity of brain regions and facial muscle reactions revealed that correlated regions are related to motor (IFG, pre-SMA, IPL) simulation of facial expressions, but also to emotional processing. Additionally, happiness display correlations with muscle responses were found in basal ganglia structures (right caudate head, bilaterally globus pallidus and putamen), nucleus accumbens and insula, while for angry displays, in the right amygdala and insula, among others.

Activations in the IFG and pre-SMA observed in our study coincide with earlier studies (Hennenlotter et al., 2005; Lee et al., 2006; Jabbi and Keysers, 2008; Likowski et al., 2012; Kircher et al., 2013) that claimed that the regions constitute a representation network for observation and imitation of emotional facial expressions (for a review, see Bastiaansen et al., 2009). For example, Lee et al. (2006), who also explored the relation between brain activity and facial muscle movement (facial markers), emphasized the role of the IFG in intentional imitation of emotional expressions.

It is interesting that the results of our study indicated that activation of the pre-SMA correlated with magnitude of facial muscle response for happy dynamic displays. Consistent with our results, similar ones were observed in a study by Iwase et al. (2002) during spontaneous facial execution of smiling. It was proposed that the activation of the pre-SMA could be understood as contagion of the happy facial expressions (Dimberg et al., 2002) due to pre-SMA connections to the striatum (Lehéricy et al., 2004), a critical component of the motor and reward systems. Moreover, it is well known that smiles evoke a positive response (Sims et al., 2012), serving as socially rewarding stimuli (Heerey and Crossley, 2013) in face-to-face interactions. This interpretation fits with our results of the involvement of the basal ganglia and nucleus accumbens, structures constituting reward-related circuitry (for a review, see Kringelbach and Berridge, 2010) in the processing of positive facial expressions. Basal nuclei activity correlated positively with ZM (nucleus accumbens, putamen) and negatively with CS (caudate head) activity for happiness dynamic displays, which is consistent with previous findings in the literature. It has been shown that the nuclei accumbens responds for different positive stimuli, such as money (Clithero et al., 2011), erotic pictures (Sabatinelli et al., 2007) or happy facial displays (Monk et al., 2008), and is thought to be involved in the experience of pleasure (Ernst et al., 2004). This interpretation is supported by the fact that for happiness displays we also found a significant EMG response in the OO muscle, which could mean that subjects recognized happiness as “real” smiles. Furthermore, our results remain in agreement with another earlier study (Likowski et al., 2012) in which the stronger ZM reactions to happy faces were associated with an increase in activity in the right caudate. This corresponds to Vrticka et al. (2013) who showed that the left putamen is more activated during imitation than passive observation of happy displays.

Interestingly, in our study, the activation in the right caudate also correlated positively with OO reactions for anger expressions. Caudate nucleus, part of the dorsal stratum, is known to be involved in motor and non-motor processes, e.g., including procedural learning, associative learning and inhibitory control of action (Soghomonian, 2016). Moreover, it is suggested that activity of the basal ganglia also reflect approach motivation and could represent reward (O'Doherty et al., 2003; Lee et al., 2006). Recently, Mühlberger et al. (2011) reported that perception of both happy and anger dynamic facial expressions were related to dorsal striatum activity. Furthermore, the activity of caudate nuclei during perception of anger may reflect a more general role in detection of danger signals. For example, it has been shown that PD subjects exhibited selective impairments in the recognition of negative facial emotion, e.g., for anger (Sprengelmeyer et al., 2003; Clark et al., 2008); fear (Livingstone et al., 2016), and sadness and disgust (Sprengelmeyer et al., 2003; Dujardin et al., 2004). Accordingly, neuroimaging data from healthy subjects tends to confirm the role of caudate nuclei in processing of negative emotions, particularly in recognition of angry expression (Beyer et al., 2017).

Importantly, we observed that during perception of anger dynamic displays, OO response correlated not only with caudate nucleus but with the right amygdala activity as well. Historically, the amygdala has been observed as playing a substantial role in the processing and expression of fear, but has recently been linked to other emotions, both positive and negative. For example, some studies have found amygdala activation during the observation and execution of both negative and positive facial expressions (Carr et al., 2003; van der Gaag et al., 2007), suggesting that this structure may reflect not only imitation but also the experience of a particular emotion (Kircher et al., 2013). As far as contraction of OO for anger expressions is concerned, this could be interpreted as a reaction to negative signal value or as a sign of arousal or interest (Witvliet and Vrana, 1995).

Further, in our study, we observed correlations between activity of the insula and facial responses during perception of both happy and angry facial expressions. Recently, a considerable number of studies (Carr et al., 2003; van der Gaag et al., 2007; Jabbi and Keysers, 2008) have suggested that the anterior insula and adjacent inferior frontal operculum (IFO) may represent an emotional component of the MNS. The role of those structures has been shown not only for observing but also for experiencing of emotions [i.e., unpleasant odors, (Wicker et al., 2003) or tastes (Jabbi et al., 2007)]. Moreover, the insula is involved in the experience of positive emotions, such as during the viewing of pleasing facial expressions (Jabbi et al., 2007), or during observation and execution of smile expressions (Hennenlotter et al., 2005). As far as the nature of FM is concerned, there is an idea that the insula and IFO may underlie a simulation of emotional feeling states (referred to as *hot simulation*). In contrast, the IFG (which activates during observation of neutral and emotional facial expressions) may reflect a form of motor simulation (referred to as *cold simulation*) (for a review, see Bastiaansen et al., 2009). The support for this idea comes from connectivity analysis of IFO and IFG activity where subjects experience unpleasant and neutral tastes. Using Granger causality, Jabbi and Keysers (2008) showed that activity in the IFO (a structure functionally related to the insula) is causally triggered by activity in the IFG. In other words, motor simulation in the IFG seems to trigger an affective simulation in the IFO of what the other person is feeling. Our results regarding the correlated activity of the IFG and muscle responses, as well as the separate correlated activity between those muscle responses and the insula, seem to be in line with the aforementioned interpretation.

It should be noted that in our study, as in others (Lee et al., 2006; van der Gaag et al., 2007), we did not observed activity in the motor or somatosensory cortex during passive viewing of emotional expressions. Indeed, there is a theoretical assumption that FM processes activate motor as well as somatosensory neuronal structures involved in processing the facial expression (Korb et al., 2015; Paracampo et al., 2016; Wood et al., 2016). Conversely, based on neuroimaging data, it seems that the magnitude of facial muscular change during emotional expression resonates activity related to emotion processing, i.e., insula or amygdala (Lee et al., 2006; van der Gaag et al., 2007), rather than the motor and somatosensory cortex. Moreover, it was shown that explicit imitation and not passive observation of facial expressions engages more somatosensory and premotor cortices. Accordingly, it was shown that activity in IFG was more pronounced during imitation than passive viewing of emotional expression (Carr et al., 2003).

In conclusion, our study confirmed the general agreement that exists among researchers that dynamic facial expressions are a valuable source of information in social communication. The evidence was visible during the stronger FM and greater neural network activations during dynamic compared to static facial expressions of happiness and anger. Moreover, the direct relationships between FM response and brain activity revealed that the associated structures belong to motor and emotional components of the FM phenomenon. The activity of the IFG and pre-SMA (classical MNS) appears to reflect action representation (i.e., the motor aspects of observed facial expressions), while the insula and amygdalae (extended MNS) process the emotional content of facial expressions. Furthermore, it seems that our results agree with the proposal that FM is not pure motor copy of behavior but rather it engages unique neural networks involved in emotion processing. Based on the current set of knowledge, it seems FM includes motor imitation and emotional contagion processes; however, their mutual relations are so far not established conclusively. For example, it could be possible that motor imitation leads to emotional contagion or vice versa, among other factors which could play an important role in social interactions.

## Author contributions

Conceived and designed the experiments: KR and ŁZ. Performed the experiments: KR and ŁZ. Analyzed the data: KR and ŁZ. Contributed materials: KR and ŁZ. Wrote the paper: KR, ŁZ, KJ-S, and IS.

### Conflict of interest statement

The authors declare that the research was conducted in the absence of any commercial or financial relationships that could be construed as a potential conflict of interest.
