# Supplementary material for: Neural Correlates of Facial Mimicry: Simultaneous Measurements of EMG and BOLD Responses during Perception of Dynamic Compared to Static Facial Expressions
Source: Front Psychol. 2018 Feb 6;9:52. doi: 10.3389/fpsyg.2018.00052 (PMC5807922; doi:10.3389/fpsyg.2018.00052)
Supplement: Supplementary file 1 [file Table1.DOCX]

Appendix presents whole brain analysis (results of flexible factorial model) of selected contrasts. All contrasts were *p* < 0.05 Family Wise Corrected (FWE), with no additional threshold for a number of voxels activated in the regional cluster (k=1). Abbreviations: L - left hemisphere; R - right hemisphere. Brain labels describing peak activations are reported using SPM Anatomy Toolbox (Eickhoff, 2016).

Supplementary Table 1 Whole brain peak activations for happiness dynamic > happiness static contrast.

| Region | side | k | x | y | z | t |
| --- | --- | --- | --- | --- | --- | --- |
| Middle Temporal Gyrus | R | 4503 | 46 | -64 | 2 | 16,08 |
| Superior Temporal Gyrus | R |  | 54 | -40 | 10 | 12,65 |
|  |  |  | 48 | -24 | -6 | 8,24 |
| Fusiform Gyrus | R |  | 44 | -44 | -16 | 7,42 |
| Middle Occipital Gyrus | L | 1879 | -48 | -72 | 2 | 11,94 |
| Precentral Gyrus | R | 724 | 50 | 4 | 46 | 8,05 |
|  |  |  | 44 | 2 | 42 | 7,71 |
| IFG (p. Opercularis) | R |  | 50 | 16 | 26 | 5,49 |
| Cerebelum (VI) | L | 185 | -14 | -74 | -24 | 6,25 |
| Cerebelum (VII) | L |  | -16 | -76 | -42 | 5,83 |
|  |  |  | -10 | -72 | -42 | 5,57 |
| Cerebelum (Crus 2) | L |  | -20 | -78 | -36 | 5,17 |
| Temporal Pole | R | 99 | 52 | 8 | -20 | 6,18 |
| Medial Temporal Pole | R |  | 48 | 12 | -26 | 5,48 |
| Inferior Parietal Lobule | R | 64 | 36 | -48 | 54 | 5,80 |
| IFG (p. Orbitalis) | R | 124 | 34 | 28 | -12 | 5,78 |
| Insula Lobe | R |  | 36 | 22 | -6 | 5,68 |
| IFG (p. Triangularis) | R | 42 | 50 | 32 | -2 | 5,51 |
| Insula Lobe | L | 9 | -36 | 18 | 2 | 5,09 |
| Pallidum | R | 12 | 16 | 6 | 4 | 5,08 |
| Cerebellar Vermis (9) |  | 7 | 0 | -54 | -38 | 4,94 |

Supplementary Table 2 Whole brain peak activations for anger dynamic > anger static contrast.

| Region | side | k | x | y | z | t |
| --- | --- | --- | --- | --- | --- | --- |
| Middle Temporal Gyrus | R | 4301 | 46 | -64 | 4 | 18,52 |
| Superior Temporal Gyrus | R |  | 52 | -38 | 10 | 10,99 |
| Middle Temporal Gyrus | R |  | 64 | -44 | 10 | 10,03 |
| Fusiform Gyrus | R |  | 44 | -46 | -16 | 9,32 |
| Inferior Temporal Gyrus | R |  | 42 | -50 | -14 | 9,03 |
| Superior Temporal Gyrus | R |  | 64 | -36 | 16 | 8,70 |
| Middle Occipital Gyrus | L | 1681 | -44 | -70 | 4 | 13,16 |
| Middle Temporal Gyrus | L |  | -50 | -50 | 8 | 7,19 |
|  |  |  | -46 | -48 | 8 | 7,11 |
| Precentral Gyrus | R | 745 | 48 | 4 | 48 | 8,44 |
|  |  |  | 46 | 16 | 20 | 6,12 |
| IFG (p. Opercularis) | R |  | 46 | 14 | 26 | 5,92 |
|  |  |  | 54 | 12 | 30 | 5,53 |
| Cerebelum (VII) | L | 90 | -16 | -76 | -42 | 6,79 |
| Cerebelum (Crus 2) | L |  | -12 | -78 | -40 | 6,67 |
| Hippocampus | R | 118 | 24 | -4 | -20 | 6,23 |
| Temporal Pole | R |  | 30 | 4 | -22 | 5,67 |
| Medial Temporal Pole | R | 108 | 52 | 6 | -20 | 5,92 |
| IFG (p. Triangularis) | R | 75 | 58 | 32 | 12 | 5,43 |
|  |  |  | 52 | 30 | 0 | 5,19 |
| Amygdala | L | 10 | -22 | -6 | -16 | 5,16 |

Supplementary Table 3 Whole brain peak activations for neutral dynamic > neutral static contrast.

| Region | side | k | x | y | z | t |
| --- | --- | --- | --- | --- | --- | --- |
| Middle Temporal Gyrus | R | 1235 | 44 | -64 | 4 | 9,33 |
|  |  |  | 54 | -40 | 4 | 6,36 |
|  |  |  | 50 | -42 | 10 | 6,20 |
| Superior Temporal Gyrus | R |  | 66 | -34 | 16 | 5,88 |
| Middle Occipital Gyrus | L | 147 | -50 | -74 | 2 | 6,34 |
| Middle Temporal Gyrus | L |  | -44 | -66 | 6 | 5,38 |
| Middle Temporal Gyrus | R | 34 | 50 | -14 | -14 | 5,52 |

Supplementary Table 4 Whole brain peak activations for emotion dynamic > emotion static contrast.

| Region | side | k | x | y | z | t |
| --- | --- | --- | --- | --- | --- | --- |
| Middle Temporal Gyrus | R | 6466 | 46 | -62 | 4 | 23,18 |
| Superior Temporal Gyrus | R |  | 54 | -40 | 10 | 15,75 |
| Fusiform Gyrus | R |  | 44 | -46 | -16 | 11,28 |
| Temporal Pole | R |  | 52 | 8 | -20 | 8,14 |
| Amygdala | R |  | 22 | -4 | -18 | 6,26 |
| Temporal Pole | R |  | 34 | 6 | -22 | 6,00 |
| Hippocampus | R |  | 30 | -12 | -12 | 5,70 |
|  |  |  | 36 | 0 | -16 | 5,54 |
|  |  |  | 34 | -4 | -14 | 5,49 |
| Middle Occipital Gyrus | L | 2581 | -44 | -70 | 4 | 16,77 |
| Precentral Gyrus | R | 1469 | 48 | 4 | 46 | 10,97 |
| IFG (p. Opercularis) | R |  | 46 | 14 | 26 | 7,50 |
| Cerebelum (VII) | L | 436 | -16 | -76 | -42 | 8,51 |
| Cerebelum (Crus 1) | L |  | -18 | -74 | -26 | 6,56 |
|  |  |  | -22 | -76 | -28 | 6,26 |
| IFG (p. Triangularis) | R | 454 | 50 | 30 | -2 | 6,94 |
|  |  |  | 52 | 32 | 8 | 5,30 |
| IFG (p. Orbitalis) | R |  | 36 | 30 | -14 | 5,21 |
| IFG (p. Triangularis) | R |  | 52 | 28 | 10 | 5,17 |
|  |  |  | 56 | 36 | 10 | 4,93 |
| Posterior-Medial Frontal | R | 43 | 10 | 14 | 66 | 5,67 |
| Fusiform Gyrus | L | 44 | -40 | -48 | -14 | 5,54 |
| Pallidum | R | 70 | 16 | 6 | 4 | 5,42 |
| Amygdala | L | 11 | -28 | 2 | -22 | 5,27 |
| Inferior Parietal Lobule | R | 19 | 32 | -48 | 50 | 5,17 |
| Cerebelum (Crus 1) | L | 12 | -44 | -64 | -26 | 5,16 |
| Amygdala | L | 7 | -22 | -6 | -14 | 5,06 |
| Pallidum | L | 3 | -10 | 4 | -2 | 4,92 |
| Superior Medial Gyrus | R | 7 | 8 | 56 | 24 | 4,83 |
|  |  | 1 | 10 | -26 | -8 | 4,77 |

Supplementary Table 5 Whole brain peak activations for all dynamic > all static contrast.

| Region | side | k | x | y | z | t |
| --- | --- | --- | --- | --- | --- | --- |
| Middle Temporal Gyrus | R | 8830 | 46 | -64 | 4 | 24,00 |
| Superior Temporal Gyrus | R |  | 54 | -40 | 10 | 16,25 |
| Fusiform Gyrus | R |  | 44 | -46 | -16 | 11,20 |
| Precentral Gyrus | R |  | 48 | 4 | 46 | 10,80 |
| IFG (p. Triangularis) | R |  | 46 | 16 | 22 | 8,66 |
| IFG (p. Opercularis) | R |  | 52 | 14 | 28 | 8,45 |
| Temporal Pole | R |  | 48 | 8 | -22 | 7,83 |
| IFG (p. Orbitalis) | R |  | 46 | 30 | -4 | 6,87 |
| IFG (p. Triangularis) | R |  | 50 | 30 | -2 | 6,81 |
|  |  |  | 36 | 0 | -16 | 6,29 |
| Temporal Pole | R |  | 34 | 6 | -22 | 6,00 |
| Hippocampus | R |  | 22 | -6 | -16 | 5,88 |
| IFG (p. Triangularis) | R |  | 52 | 28 | 10 | 5,24 |
| Hippocampus | R |  | 30 | -10 | -14 | 5,13 |
| Middle Occipital Gyrus | L | 2480 | -48 | -72 | 2 | 16,99 |
| Middle Temporal Gyrus | L |  | -48 | -48 | 8 | 10,41 |
| Superior Temporal Gyrus | L |  | -58 | -38 | 20 | 5,03 |
| Cerebelum (VII) | L | 487 | -16 | -76 | -42 | 8,89 |
| Cerebelum (Crus 1) | L |  | -18 | -70 | -28 | 6,00 |
| Fusiform Gyrus | L | 56 | -40 | -48 | -14 | 5,57 |
| Inferior Temporal Gyrus | L |  | -42 | -48 | -18 | 5,46 |
| Pallidum | R | 77 | 16 | 6 | 0 | 5,54 |
|  |  |  | 20 | 2 | 8 | 5,18 |
| Posterior-Medial Frontal | R | 62 | 10 | 14 | 64 | 5,53 |
| Inferior Parietal Lobule | R | 55 | 34 | -48 | 52 | 5,47 |
|  |  | 3 | 12 | -26 | -10 | 5,02 |
| Amygdala | L | 1 | -28 | 2 | -22 | 4,79 |
| Cerebelum (Crus 1) | L | 1 | -44 | -64 | -26 | 4,77 |

Supplementary Table 6 Whole brain peak activations for happiness dynamic > neutral dynamic contrast.

| Region | side | k | x | y | z | t |
| --- | --- | --- | --- | --- | --- | --- |
| Middle Occipital Gyrus | L | 3088 | -26 | -92 | 2 | 10,73 |
| Inferior Occipital Gyrus | L |  | -46 | -72 | -8 | 8,36 |
| Fusiform Gyrus | L |  | -40 | -74 | -14 | 8,01 |
| Cerebelum (VI) | L |  | -30 | -62 | -22 | 6,26 |
| Cerebelum (Crus 2) | L |  | -28 | -80 | -38 | 5,96 |
| Middle Temporal Gyrus | L |  | -54 | -64 | 8 | 5,52 |
|  |  |  | -50 | -60 | 8 | 5,42 |
| Cerebelum (Crus 1) | L |  | -42 | -56 | -28 | 5,36 |
|  |  |  | -40 | -56 | 6 | 5,08 |
| Middle Occipital Gyrus | R | 3412 | 30 | -88 | 4 | 9,51 |
| Middle Temporal Gyrus | R |  | 56 | -64 | 0 | 8,59 |
| Superior Occipital Gyrus | R |  | 20 | -100 | 6 | 7,99 |
| Middle Temporal Gyrus | R |  | 50 | -34 | 0 | 7,30 |
| Inferior Occipital Gyrus | R |  | 44 | -76 | -8 | 7,10 |
| Cerebelum (VI) | R |  | 36 | -58 | -24 | 6,73 |
|  |  |  | 48 | -48 | 4 | 6,61 |
| Fusiform Gyrus | R |  | 44 | -64 | -18 | 6,38 |
|  |  |  | 42 | -44 | -16 | 6,37 |
|  |  |  | 44 | -26 | -6 | 5,96 |
| Cerebelum (Crus 1) | R |  | 28 | -82 | -24 | 4,84 |
| Temporal Pole | L | 498 | -36 | 20 | -28 | 7,92 |
|  |  |  | -42 | 22 | -16 | 5,85 |
| Area 45 | L |  | -54 | 20 | -6 | 5,54 |
| Superior Medial Gyrus | L | 2421 | -2 | 58 | 22 | 7,23 |
| Middle Frontal Gyrus | L |  | -20 | 48 | 32 | 7,02 |
| Superior Medial Gyrus | R |  | 6 | 56 | 24 | 7,02 |
| Superior Frontal Gyrus | R |  | 18 | 54 | 28 | 6,84 |
| Middle Frontal Gyrus | L |  | -22 | 52 | 26 | 6,62 |
| Superior Medial Gyrus | L |  | -10 | 26 | 34 | 6,39 |
|  |  |  | -8 | 32 | 30 | 6,32 |
| ACC | L |  | -2 | 52 | 4 | 6,10 |
| Superior Medial Gyrus | R |  | 4 | 50 | 4 | 5,97 |
| ACC | L |  | -4 | 38 | 20 | 5,37 |
|  |  |  | -6 | 34 | 12 | 5,24 |
| Medial Temporal Pole | R | 308 | 50 | 10 | -30 | 6,84 |
|  |  |  | 48 | 8 | -34 | 6,49 |
| Inferior Temporal Gyrus | R |  | 46 | 6 | -38 | 6,42 |
| Medial Temporal Pole | R |  | 42 | 20 | -36 | 5,25 |
|  |  |  | 44 | 24 | -34 | 5,24 |
| IFG (p. Orbitalis) | R | 200 | 34 | 28 | -16 | 6,77 |
| Insula Lobe | R |  | 30 | 20 | -14 | 6,14 |
| Hippocampus | L | 225 | -18 | -10 | -12 | 6,74 |
| Amygdala | L |  | -20 | 2 | -16 | 5,24 |
|  |  |  | -2 | -16 | -16 | 4,93 |
| Thal: Parietal | L |  | -26 | -18 | -8 | 4,85 |
| Posterior-Medial Frontal | R | 159 | 8 | 16 | 62 | 6,63 |
|  |  | 202 | 12 | 8 | 4 | 6,18 |
| Caudate Nucleus | R |  | 10 | 6 | 12 | 5,71 |
| Pallidum | L | 250 | -12 | 4 | 0 | 6,03 |
| Caudate Nucleus | L |  | -8 | 6 | 14 | 5,77 |
| Thal: Prefrontal | L |  | -12 | -4 | 12 | 5,28 |
| PCC | L | 285 | -2 | -46 | 20 | 5,80 |
| Precuneus | L |  | -4 | -56 | 34 | 5,45 |
| Inferior Parietal Lobule | L | 159 | -50 | -60 | 44 | 5,75 |
| Angular Gyrus | L |  | -46 | -54 | 26 | 5,02 |
| Cerebelum (Crus 2) | R | 85 | 34 | -72 | -38 | 5,68 |
| Temporal Pole | R | 13 | 34 | 8 | -22 | 5,54 |
| Thal: Temporal | R | 25 | 30 | -18 | -8 | 5,50 |
| Precentral Gyrus | R | 19 | 52 | 4 | 46 | 5,45 |
| Superior Frontal Gyrus | L | 36 | -12 | 36 | 50 | 5,41 |
| SupraMarginal Gyrus | R | 193 | 62 | -22 | 24 | 5,41 |
|  |  |  | 64 | -16 | 26 | 5,34 |
| Postcentral Gyrus | R |  | 64 | -14 | 36 | 5,10 |
| IFG (p. Triangularis) | R | 61 | 48 | 28 | -2 | 5,37 |
|  |  |  | 56 | 24 | -2 | 5,14 |
| IFG (p. Orbitalis) | R |  | 52 | 24 | -8 | 5,05 |
| IFG (p. Opercularis) | R |  | 58 | 16 | -2 | 4,82 |
| Cerebelum (IX) | R | 23 | 4 | -52 | -42 | 5,28 |
|  |  | 17 | 4 | -36 | -44 | 5,21 |
|  |  | 15 | -30 | 14 | -2 | 5,18 |
| Thal: Visual | R | 6 | 12 | -30 | -4 | 5,05 |
|  |  | 9 | -12 | -30 | -8 | 5,04 |
|  |  | 8 | 16 | -18 | -14 | 4,98 |
| Middle Frontal Gyrus | L | 4 | -44 | 14 | 44 | 4,94 |
| Medial Temporal Pole | L | 8 | -52 | 10 | -36 | 4,91 |
| Fusiform Gyrus | L | 1 | -42 | -48 | -22 | 4,88 |
| Thal: Temporal | L | 1 | -30 | -18 | -8 | 4,86 |
| Superior Medial Gyrus | L | 1 | -6 | 24 | 62 | 4,84 |
| Hippocampus | R | 1 | 30 | -4 | -26 | 4,80 |
| IFG (p. Orbitalis) | R | 1 | 34 | 40 | -20 | 4,78 |
| Thalamus | L | 1 | -16 | -12 | 14 | 4,78 |

Supplementary Table 7 Whole brain peak activations for happiness static > neutral static contrast.

| Region | side | k | x | y | z | t |
| --- | --- | --- | --- | --- | --- | --- |
| Superior Medial Gyrus | L | 527 | -10 | 56 | 16 | 6,33 |
|  |  |  | -4 | 56 | 22 | 6,18 |
|  |  |  | -6 | 50 | 34 | 5,54 |
| Middle Occipital Gyrus | R | 153 | 28 | -92 | 4 | 6,17 |
| Temporal Pole | L | 110 | -48 | 24 | -16 | 6,12 |
|  |  |  | -42 | 32 | -20 | 5,31 |
| Middle Occipital Gyrus | L | 226 | -26 | -92 | 4 | 6,03 |
|  |  |  | -18 | -100 | 2 | 5,63 |
| Hippocampus | L | 4 | -30 | -36 | -6 | 5,23 |
| Superior Frontal Gyrus | R | 8 | 16 | 56 | 34 | 5,18 |
| Superior Medial Gyrus | L | 14 | -6 | 42 | 50 | 5,12 |
| Angular Gyrus | L | 26 | -48 | -62 | 30 | 5,09 |
| Medial Temporal Pole | R | 2 | 50 | 16 | -38 | 5,06 |
| Superior Frontal Gyrus | L | 9 | -16 | 30 | 40 | 5,06 |
| Medial Temporal Pole | R | 6 | 50 | 18 | -34 | 5,04 |
| Middle Frontal Gyrus | L | 6 | -44 | 20 | 44 | 4,97 |
| Fusiform Gyrus | L | 8 | -44 | -70 | -18 | 4,94 |
| Cerebelum (Crus 2) | L | 1 | -26 | -76 | -40 | 4,86 |
| Precuneus | L | 5 | -2 | -52 | 34 | 4,85 |
| Inferior Temporal Gyrus | R | 1 | 54 | 4 | -36 | 4,81 |
| Inferior Temporal Gyrus | R | 2 | 48 | 8 | -40 | 4,81 |
|  |  | 2 | -4 | 2 | 14 | 4,81 |
| Inferior Temporal Gyrus | R | 1 | 52 | 6 | -38 | 4,79 |
| Medial Temporal Pole | R | 1 | 42 | 24 | -36 | 4,76 |

Supplementary Table 8 Whole brain peak activations for anger dynamic > neutral dynamic contrast.

| Region | side | k | x | y | z | t |
| --- | --- | --- | --- | --- | --- | --- |
| Middle Temporal Gyrus | R | 2750 | 56 | -64 | 2 | 10,52 |
|  |  |  | 46 | -52 | 6 | 9,41 |
| Middle Temporal Gyrus | R |  | 50 | -36 | 2 | 8,78 |
|  |  |  | 44 | -26 | -6 | 6,66 |
| Superior Temporal Gyrus | R |  | 68 | -28 | 22 | 6,22 |
| Area hOc4lp | R |  | 30 | -84 | -2 | 5,71 |
| Middle Occipital Gyrus | R |  | 38 | -90 | 4 | 5,04 |
| Middle Temporal Gyrus | L | 1634 | -54 | -64 | 8 | 8,54 |
|  |  |  | -52 | -68 | 8 | 8,45 |
| Middle Occipital Gyrus | L |  | -44 | -74 | 2 | 7,84 |
| Inferior Occipital Gyrus | L |  | -34 | -86 | -4 | 6,59 |
| Middle Temporal Gyrus | L |  | -52 | -48 | 4 | 5,90 |
| Fusiform Gyrus | R | 88 | 42 | -44 | -16 | 6,52 |
| Posterior-Medial Frontal | R | 82 | 8 | 16 | 62 | 6,29 |
| Temporal Pole | R | 80 | 54 | 14 | -22 | 5,91 |
| Medial Temporal Pole | R |  | 50 | 18 | -28 | 5,11 |
| IFG (p. Triangularis) | R | 98 | 52 | 30 | 0 | 5,81 |
| Cerebelum (Crus 2) | L | 86 | -26 | -82 | -38 | 5,75 |
| Precentral Gyrus | R | 38 | 52 | 6 | 48 | 5,55 |
| Temporal Pole | L | 45 | -38 | 22 | -20 | 5,47 |
| Superior Medial Gyrus | R | 47 | 6 | 56 | 26 | 5,44 |
| Hippocampus | L | 20 | -20 | -8 | -12 | 5,37 |
| Superior Frontal Gyrus | R | 17 | 18 | 58 | 28 | 5,21 |
| Temporal Pole | L | 8 | -32 | 8 | -20 | 5,14 |
| Inferior Temporal Gyrus | R | 9 | 46 | 6 | -38 | 5,08 |
| Temporal Pole | R | 11 | 32 | 6 | -22 | 5,06 |
| Thal: Temporal | R | 3 | 32 | -16 | -8 | 4,88 |
| Cerebelum (Crus 1) | L | 1 | -22 | -74 | -26 | 4,81 |
| Amygdala | R | 1 | 26 | -2 | -22 | 4,78 |

Supplementary Table 9 Whole brain peak activations for anger static > neutral static contrast.

No significant activations.

Supplementary Table 10 Whole brain peak activations for emotion dynamic > neutral dynamic contrast.

| Region | side | k | x | y | z | t |
| --- | --- | --- | --- | --- | --- | --- |
| Middle Temporal Gyrus | R | 4127 | 56 | -64 | 0 | 10,80 |
|  |  |  | 50 | -36 | 2 | 9,07 |
|  |  |  | 46 | -50 | 6 | 9,03 |
| Middle Occipital Gyrus | R |  | 32 | -86 | 2 | 8,06 |
| Fusiform Gyrus | R |  | 42 | -44 | -16 | 7,36 |
|  |  |  | 44 | -26 | -6 | 7,21 |
| Superior Occipital Gyrus | R |  | 22 | -100 | 8 | 6,31 |
| SupraMarginal Gyrus | R |  | 62 | -24 | 22 | 6,14 |
|  |  |  | 58 | -26 | 24 | 5,92 |
| Cerebelum (Crus 1) | R |  | 42 | -68 | -22 | 5,66 |
| Postcentral Gyrus | R |  | 64 | -16 | 38 | 5,49 |
| Cerebelum (VI) | R |  | 34 | -62 | -24 | 5,36 |
| Postcentral Gyrus | R |  | 66 | -16 | 30 | 5,07 |
| Middle Occipital Gyrus | L | 3194 | -28 | -92 | 2 | 9,18 |
| Inferior Occipital Gyrus | L |  | -48 | -72 | -6 | 8,48 |
| Middle Temporal Gyrus | L |  | -50 | -70 | 0 | 8,46 |
| Middle Occipital Gyrus | L |  | -44 | -72 | 0 | 8,44 |
| Middle Temporal Gyrus | L |  | -54 | -64 | 8 | 8,05 |
|  |  |  | -52 | -68 | 8 | 8,01 |
| Cerebelum (Crus 2) | L |  | -28 | -80 | -38 | 6,68 |
| Middle Temporal Gyrus | L |  | -52 | -48 | 4 | 5,88 |
| Cerebelum (Crus 1) | L |  | -26 | -74 | -26 | 5,66 |
|  |  |  | -32 | -80 | -28 | 5,30 |
| Posterior-Medial Frontal | R | 202 | 8 | 16 | 62 | 7,37 |
| Superior Medial Gyrus | R | 961 | 6 | 58 | 26 | 7,05 |
| Superior Frontal Gyrus | R |  | 18 | 58 | 28 | 6,78 |
| Superior Medial Gyrus | L |  | 2 | 56 | 26 | 6,78 |
| Middle Frontal Gyrus | L |  | -22 | 52 | 28 | 5,77 |
| Temporal Pole | L | 465 | -38 | 20 | -26 | 6,94 |
| IFG (p. Orbitalis) | L |  | -34 | 20 | -22 | 6,88 |
| Hippocampus | L |  | -20 | -8 | -12 | 6,69 |
| Temporal Pole | L |  | -32 | 8 | -20 | 5,21 |
|  |  |  | -4 | -16 | -14 | 5,03 |
| Amygdala | L |  | -26 | 2 | -20 | 4,97 |
|  |  |  | -8 | -14 | -14 | 4,94 |
| Amygdala | L |  | -22 | 2 | -18 | 4,90 |
| Medial Temporal Pole | R | 396 | 50 | 10 | -30 | 6,66 |
| Inferior Temporal Gyrus | R |  | 46 | 6 | -38 | 6,56 |
| Temporal Pole | R |  | 54 | 16 | -22 | 6,49 |
| Medial Temporal Pole | R |  | 48 | 8 | -34 | 6,47 |
| IFG (p. Orbitalis) | R |  | 36 | 26 | -16 | 5,47 |
| Insula Lobe | R |  | 30 | 20 | -16 | 5,43 |
| IFG (p. Orbitalis) | R |  | 44 | 22 | -18 | 5,09 |
| IFG (p. Triangularis) | R | 168 | 50 | 28 | -2 | 6,34 |
| Precentral Gyrus | R | 68 | 52 | 4 | 46 | 6,25 |
| Temporal Pole | R | 53 | 34 | 10 | -22 | 5,99 |
| Hippocampus | R |  | 26 | -2 | -24 | 5,01 |
| Thal: Temporal | R | 35 | 32 | -16 | -8 | 5,83 |
|  |  | 63 | 0 | -48 | 20 | 5,48 |
|  |  | 54 | 12 | 8 | 4 | 5,38 |
| Caudate Nucleus | R |  | 10 | 8 | 12 | 5,07 |
| Pallidum | L | 75 | -12 | 4 | 0 | 5,30 |
| Caudate Nucleus | L |  | -10 | 6 | 12 | 5,24 |
| Thal: Prefrontal | L |  | -14 | -2 | 10 | 4,78 |
| Inferior Temporal Gyrus | L | 14 | -44 | -42 | -16 | 5,15 |
| IFG (p. Orbitalis) | L | 19 | -52 | 20 | -6 | 5,14 |
| Superior Medial Gyrus | L | 4 | -10 | 32 | 32 | 4,82 |
| Medial Temporal Pole | L | 1 | -50 | 12 | -36 | 4,76 |

Supplementary Table 11 Whole brain peak activations for emotion static > neutral static contrast.

| Region | side | k | x | y | z | t |
| --- | --- | --- | --- | --- | --- | --- |
| Medial Temporal Pole | R | 11 | 50 | 20 | -32 | 5,24 |
| Cerebelum (Crus 2) | L | 2 | -26 | -76 | -40 | 4,95 |
| Posterior-Medial Frontal | R | 6 | 8 | 14 | 62 | 4,92 |
